# NFAT5: a stress-related transcription factor with multiple functions in health and disease

**DOI:** 10.15698/cst2025.05.304

**Published:** 2025-05-22

**Authors:** Alfredo Domínguez-López, Fátima S. Magaña-Guerrero, Beatriz Buentello-Volante, Óscar Vivanco-Rojas, Yonathan Garfias

**Affiliations:** 1 Department of Biochemistry, Faculty of Medicine, Universidad Nacional Autónoma de México. Mexico City, Mexico. 04510.; 2 Cell and Tissue Biology Department, Research Unit, Institute of Ophthalmology Conde de Valenciana. Mexico City, Mexico. 06800.

**Keywords:** NFAT5, TonEBP, transcription factor, osmolality, cell stress, immune response

## Abstract

Nuclear factor of activated T cells 5 (NFAT5) is a transcription factor within the Rel family, primarily recognized for its role in cellular adaptation to osmotic stress, particularly in hypertonic and hyperosmotic environments. Beyond osmotic regulation, NFAT5 responds to diverse stimuli, including cytokines, growth factors, oxidative stress, and microbial signals. This versatility enables NFAT5 to regulate essential cellular processes such as proliferation, survival, migration, and vascular remodelling. In the immune system, NFAT5 modulates the function of monocytes, macrophages, astrocytes, microglia, and T cells, contributing to immune homeostasis and inflammatory responses. Dysregulation of NFAT5 activity is implicated in various pathological conditions, including autoimmune diseases, cancer, and cardiovascular disorders, largely due to its ability to control genes involved in inflammatory and immune pathways under both isotonic and hypertonic conditions. Recent studies have unveiled new regulatory mechanisms, including interactions with non-coding RNAs, offering deeper insights into the functional landscape of NFAT5 and its therapeutic potential. This review delves into the multifaceted roles of NFAT5 in health and disease, emphasizing its emerging importance as a promising therapeutic target.

## Abbreviations

AMD - age-related macular degeneration,

APC - antigen-presenting cell,

BBB - blood-brain barrier,

CLL - chronic lymphocytic leukaemia,

CNS - central nervous system,

DBD - DNA-binding domain,

DED - dry eye disease,

DMD - Duchenne muscular dystrophy,

DN - diabetic nephropathy,

DR - diabetic retinopathy,

EGFR - epidermal growth factor receptor,

eNOS - endothelial NO synthase,

ETBF - Enterotoxigenic Bacteroides fragilis,

FLS - fibroblast-like synoviocytes,

GBM - glioblastoma multiforme,

HCC - hepatocellular carcinoma,

HBV - hepatitis B virus,

HCV - hepatitis C virus,

HUVEC - human umbilical vein endothelial cells,

ICH - intracerebral haemorrhage,

iNOS - inducible NO synthase,

LN - lupus nephritis,

LPS - lipopolysaccharide,

LSCC - laryngeal squamous cell carcinoma,

LUAD - lung adenocarcinoma,

LUSC - lung squamous cell carcinoma,

mBregs - memory B regulatory cells,

MCD - medullary collecting duct,

MG - myasthenia gravis,

NLS - nuclear localization signal,

NO - nitric oxide,

NP - nucleus pulposus,

NSCLC - non-small cell lung cancer,

OA - osteoarthritis,

ODS - oral squamous cell carcinoma,

ORE - osmotic response element,

PBMCs - peripheral blood mononuclear cells,

RA - rheumatoid arthritis,

RGC - retinal ganglion cell,

RPE - retinal pigment epithelial cells,

ROS - reactive oxygen species,

SCTR - secretin receptor,

SLE - systemic lupus erythematosus,

SMIT - sodium-myo-inositol transporter,

T1DM - Type 1 Diabetes mellitus,

T2DM - Type 2 Diabetes mellitus,

TF - transcription factor,

TLR - Toll-like receptor,

VSMCs - vascular smooth muscle cells.

## INTRODUCTION

Cell homeostasis is maintained by numerous intrinsic and extrinsic factors, among which transcription factors (TFs) play a crucial role in driving cell activation or inhibition. The Rel/NFκB family includes a diverse group of TFs, such as nuclear factor kappa B (NFκB) and nuclear factor of activated T cells (NFAT1-5). Members of this family share key biochemical features, including a DNA recognition site, a calcineurin-binding site, and a calcium-dependent activation domain. Although NFAT5 belongs to the Rel family, it stands out due to its lack of a calcium-dependent activation site, resulting in a distinct functional profile compared to other members. NFAT5, also known as tonicity enhancer binding protein (TonEBP), plays a pivotal role in regulating cellular homeostasis during osmotic stress. It was initially identified in kidney medullary cells, where it controls the dramatic solute fluctuations essential for the organ osmoregulatory function. Interestingly, NFAT5 activation is not limited to hypertonic stress; it can also be triggered by isotonic stimuli, suggesting that this TF fulfils diverse functions depending on the cellular environment. Thus, NFAT5 is characterized as both a stress-responsive protein and a key regulator of hypertonic stress adaptation 1.

## ROLE OF NFAT5 IN HEALTH

### Molecular structure and DNA binding

NFAT5 was cloned in 1999, and its N-terminus was found to share significant similarity with the Rel-like DNA binding domain (DBD) of the NFAT TF family. However, unlike other NFAT isoforms, NFAT5 lacks the highly conserved N-terminal region that serves as a calcineurin-binding site 2. Additionally, its DBD differs from that of NFAT1-4, preventing cooperation with Fos/Jun at NFAT: activator protein-1 (AP-1) composite sites 3. Despite this structural divergence, pharmacological inhibition of AP-1 reduces NaCl-induced NFAT5 expression in retinal pigment epithelial (RPE) cells, suggesting that AP-1 partially mediates this response 4. This could be explained by the fact that, while NFAT1-4 recognize a broader consensus sequence (GGAAA), NFAT5 binds to a more specific motif (TGGAAA). This partial overlap may allow shared gene regulation under specific conditions, despite differences in their DBDs and regulatory pathways 3.

**Figure 1:**
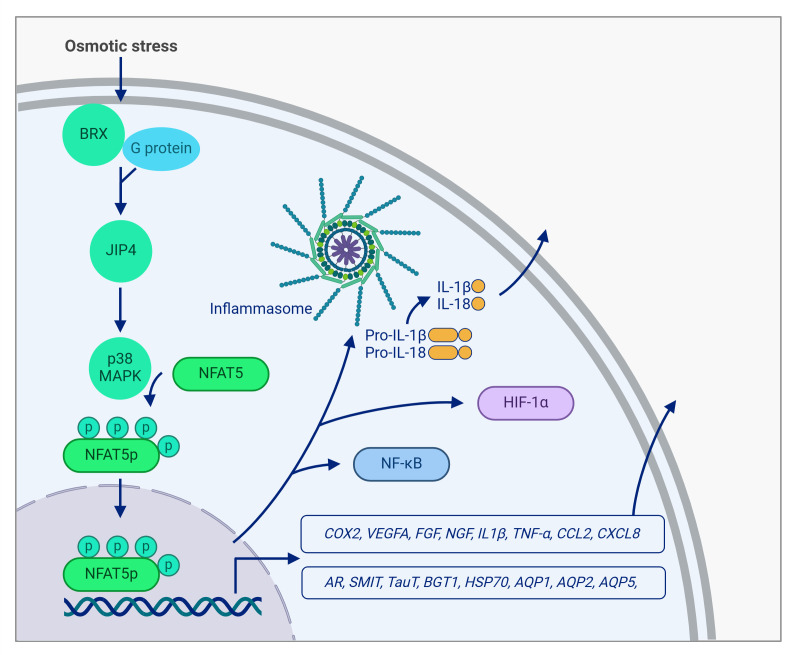
FIGURE 1: NFAT5 pathway. Osmotic stress promotes the activation of protein kinase A-anchoring protein 13 (BRX) and G proteins to stimulate c-Jun N-terminal kinase (JNK)-interacting protein 4 (JIP4), which triggers the NFAT5 phosphorylation (NFAT5p) through p38 MAPK. Nuclear translocation of NFAT5p induces the transcription of aldose reductase (AR), sodium-myo-inositol transporter (SMIT), taurine transporter (TauT), betaine transporter (BGT1), heat shock protein 70 (HSP70), cyclooxygenase-2 (COX2), vascular endothelial growth factor A (VEGF-A), fibroblast growth factor (FGF), nerve growth factor (NGF), aquaporin (AQP)-1, 2 and 5, interleukin-1 beta (IL-1β), tumor necrosis factor alpha (TNF-α), chemokine (CC motif) ligand 2 (CCL2), chemokine (CXC motif) ligand 8 (CXCL8) among others. Moreover, NFAT5p nuclear translocation induces activation of the NLRP3 inflammasome complex leading to the maturation of IL-1β and IL-18 and the expression of nuclear factor-kB (NF-κB) and hypoxia-inducible factor (HIF)-1α. Figure created with BioRender.

In response to hypertonic stress, cells synthesize osmoprotective molecules such as myo-inositol, betaine, taurine, and sorbitol to counteract the harmful effects of hyperosmolarity. The transport and synthesis of these molecules require specific proteins, including aldose reductase (AR), which converts glucose into sorbitol; the betaine transporter (BGT1); the sodium-myo-inositol transporter (SMIT); and the taurine transporter (TauT). Beyond osmoprotection, NFAT5 also regulates the expression of innate immune cytokines such as tumor necrosis factor-alpha (TNF-α) and lymphotoxin-β (LTβ), contributing to T cell activation 5. **Figure 1**

Upstream in the NFAT5 signalling cascade, Brx (protein kinase A-anchoring protein 13 (AKAP13)) activates specific G proteins via its guanine nucleotide exchange factor domains, facilitating the recruitment of c-Jun N-terminal kinase (JNK)-interacting protein 4 (JIP4). This, in turn, stimulates the p38 activation cascade necessary for NFAT5 expression 6. NFAT5 activation is a highly regulated process involving multiple signalling pathways, including p38, Fyn, protein kinase A (PKA), ataxia telangiectasia-mutated kinase (ATM), phospholipase C gamma 1 (PLCγ1), and protein kinase C α (PKCα), acting via extracellular signal-regulated kinase (ERK1/2) 7,8,9,10,11. Interestingly, while p38 plays a central role in NFAT5 activation, its inactivation occurs independently of its cognate phosphatase, mitogen-activated phosphokinase (MAPK) phosphatase-1 (MKP-1) 12. Moreover, Rac1 can activate NFAT5 through p38-independent PLCγ1 signalling 13, highlighting the existence of alternative activation routes. High NaCl concentrations further enhance NFAT5 activation by stimulating PKA, phosphatidylinositol-3-phosphate kinase (PI3K), and protein kinase B (AKT1), which phosphorylate glycogen synthase kinase-3-β-S9 (GSK-3β-S9). This phosphorylation neutralizes the inhibitory effect of GSK-3β on NFAT5, further promoting its activity 14.

NFAT5 continuously shuttles between the cytoplasm and nucleus in response to tonicity changes, a process regulated by its nuclear localization signal (NLS) and nuclear export sequence (NES) in the N-terminus 15. Nuclear import occurs via the nuclear pore complex through karyopherin β1 (KPNB1), which interacts with the NLS, while nuclear export is facilitated by the export-T (XPOT) protein 16. Additionally, nucleoporin 88 (Nup88) increases in response to hypertonic stress, retaining NFAT5 in the nucleus and enhancing the transcription of osmoprotective genes in kidney cells 17. A key contact site for DNA binding, NFAT5-T298, is crucial for nuclear translocation, functioning independently of Nup88 18. Under osmotic stress, NFAT5 isoform a (NFAT5a) can enter the nucleus despite its lipid anchoring sites, a process modulated by reversible palmitoylation 19. In HEK293 cells, rapid nuclear translocation depends on cyclin-dependent kinase-5 (CDK5) activity, which phosphorylates NFAT5 at Thr135 20. Structurally, NFAT5 possesses a transcriptional activation domain (TAD) in its C-terminal region, which is activated upon hypertonic exposure in a PKA-dependent but cAMP-independent manner 21,22. Additionally, integrin α1β1 plays a crucial role in NFAT5 activation within inner medullary collecting duct (MCD) cells, underscoring its importance in renal development 23.

As a TF, NFAT5 binds to conserved sequences to regulate gene expression, promoting the transcription of both mRNA and protein. Its 5'-TGGAAA-3' motif is relatively short and widely distributed throughout the genome, enabling NFAT5 to target a broad array of genes. This extensive regulatory network highlights NFAT5’s central role in cellular stress responses, immune modulation, and potentially, disease pathogenesis.

### Activation pathways

#### Osmotic stimuli

Osmotic regulation is fundamental for maintaining cellular, tissue, and organ function, ensuring overall homeostasis. In fluctuating osmotic environments, cells must adapt to prevent damage from excessive swelling or shrinking. NFAT5 plays a central role in this adaptive process by responding to changes in osmolality, particularly under hypertonic conditions. To counteract osmotic stress, cells upregulate osmocompensatory genes that encode proteins responsible for increasing intracellular osmolytes. Among these, NFAT5 regulates key players such as urea transporter 1 (UTA-1), which facilitates urea transport and is influenced by vasopressin, HSP70-2, a molecular chaperone that protects against apoptosis 24,25, and TauT, which is involved in taurine transport 26.

Unlike NFAT1-4, which require calcium-dependent activation, NFAT5 responds directly to hypertonic stress by translocating to the nucleus, where it binds specific promoter sequences such as the -76 GGAAA consensus site and the κ3 site within the TNF promoter in murine fibroblasts 27. This activation underscores its role in driving proinflammatory responses under hyperosmotic conditions. Additionally, high sodium chloride levels stabilize NFAT5 mRNA via its 5′-UTR, leading to a rapid yet transient increase in protein synthesis, as observed in mouse MCD cells 28. Increased intracellular ionic strength further enhances the stability of the NFAT5 N-terminal domain, promoting its interaction with the prosurvival high mobility group protein HMGI-C, which improves cell resilience to hypertonic stress 29. In this context, exon 8 of NFAT5 appears crucial for promoting TNF-α expression under these conditions 30.

At the transcriptional level, NFAT5 collaborates with other stress response pathways. For instance, it shares a common promoter site with Nrf2 in the multiple stress response region of the 5′-flanking region of the AR gene, demonstrating synergistic activity in HepG2 cells. This interaction, however, is abolished by c-Jun activity 31. Furthermore, NFAT5 contributes to metabolic adaptations under osmotic stress, as evidenced by its role in upregulating CYP2E1 32 and CYP3A 33, two hepatic enzymes whose expression is influenced by plasma osmolality. In neuropathic rat models, endothelin-1 (ET1) activates NFAT5 through the ET-1 receptor A (ETAR) 34.

NFAT5 also regulates inflammatory mediators in response to hypertonic stress. For example, it is required for COX-2 induction, as NFAT5 gene disruption prevents its upregulation under hyperosmotic conditions 35. In RPE cells, NFAT5 expression is driven by autocrine purinergic signalling, which involves ATP release, nucleoside transporter-mediated adenosine release, and the activation of P2X7, P2Y1, P2Y2, and adenosine A1 receptors 36. Additionally, NFAT5 modulates glucagon-like peptide-1 (GLP-1) secretion in response to sodium levels, as inhibition of NFAT5 in human L-cells blocks the effect of gastrin-releasing peptide (GRP) on sodium-induced GLP-1 upregulation 37. NFAT5 is implicated in nephrogenic diabetes insipidus by controlling the expression of critical genes involved in renal water reabsorption and urine concentration 38. Metalloproteinase-dependent activation of the epidermal growth factor receptor (EGFR) enhances NFAT5 activity under hypertonic conditions 39. Additionally, high sodium chloride environments induce reactive oxygen species (ROS)-dependent NFAT5 transactivation, increasing BGT1 mRNA levels in human embryonic kidney cells 40.

Evidence also suggests that aquaporin (AQP)2 expression is inhibited in the epithelial cells of the renal collecting tubules in transgenic mice overexpressing a dominant-negative form of NFAT5 41, indicating that NFAT5 directly regulates AQP2, at least in mouse kidney tissue 42. Similarly, AQP1 and AQP5 expression in nucleus pulposus (NP) cells depends on NFAT5 under hyperosmotic conditions 43. Interestingly, NFκB acts as a transcriptional repressor of AQP2, with its p65, p50, and p52 subunits binding the first 2.1 kbp of the AQP2 promoter under isotonic conditions 44. NFAT5 further regulates AQP1 expression in inner renal medullary cells 45 and AQP4 expression in astrocytes 46 from hyperosmotic-injured rat hippocampi by binding to a promoter site located between -49 and -38 bp of the AQP4 gene 47.

Thus, NFAT5 not only controls osmolyte regulation in response to hyperosmotic stress but also modulates genes involved in water transport and inflammation, reinforcing its role as a key transcriptional regulator in osmotic adaptation and cellular homeostasis.

#### Non-osmotic stimuli

Beyond osmotic stress, NFAT5 can be activated by proinflammatory cytokines. These cytokines are among the most extensively studied activators of NFAT5 during inflammatory processes. Their involvement suggests that NFAT5 plays a role in immune responses and inflammation, in addition to its known function in adaptation to osmotic stress. Once activated, NFAT5 regulates gene expression related to cellular adaptation, inflammation, and immune responses, positioning it as a key regulator of inflammatory pathways. For a more detailed review of osmosis-independent stimuli, see Halterman JA 48. In this context, SMIT regulated by NFTA5, is also activated in fibroblasts cultured under isotonic conditions depleted of neutral amino acids 49.

A striking example of NFAT5 activation in non-osmotic conditions occurs in placental hypoxia, where HIF-1α upregulates both NFAT5 and HSP70. Notably, NFAT5 reinforces this pathway by increasing HSP70 transcription, suggesting a positive feedback loop 50. This mechanism has led to the proposal of NFAT5 as a biomarker for placental hypoxia and ischemia 51, further supporting its involvement in preeclampsia 52. In trophoblasts, NFAT5 levels increase under high-calcium conditions, linking sodium availability to proangiogenic responses. This pathway involves osmotic gradients affecting cytoskeletal signalling, which is crucial for trophoblast function. Inhibiting Na^⁺^/K^⁺^-ATPase or activating it with mannitol triggers NFAT5 activation, whereas cytoskeletal disruption prevents this response. These findings suggest that impaired placental salt availability in preeclampsia could contribute to vascular dysfunction and systemic complications 53. For an extensive review of NFAT5 activation see 54.

NFAT5 also plays a protective role in hypoxia-induced cellular responses. In mouse embryonic fibroblasts (MEFs) exposed to hypoxia, NFAT5 upregulation leads to the expression of inducible nitric oxide synthase (iNOS), AQP1, and UTA-1, suggesting a role in cellular adaptation under oxygen-deprived conditions 55. Additionally, NFAT5 mediates endothelial responses to hypoxia, where it regulates HIF-1α-driven platelet-derived growth factor B (PDGFB) expression. This protective mechanism may help reduce vascular resistance and pulmonary hypertension, thereby mitigating organ dysfunction 56.

Hyperphosphatemia induces NFAT5 expression, which subsequently activates the calcium channel ORAI and its activator stromal interaction molecule (STIM), supporting calcium influx in megakaryocytes 57. In HepG2 cells, uric acid triggers the NFAT5-AR axis in an oxidative stress milieu, a mechanism relevant to the pathophysiology of non-alcoholic fatty liver disease (NAFLD) 58. Furthermore, macrophages subjected to compressive strain forces upregulate NFAT5 and proinflammatory cytokines, underscoring its role in mechanical stress responses, such as during orthodontic tooth movement 59. Interestingly, NFAT5 activation can be modulated pharmacologically. Lithium, a drug commonly used for bipolar disorder, has contrasting effects on NFAT5 in renal cells depending on exposure duration. In short-term isosmotic conditions, lithium activates NFAT5 via GSK-3β inhibition, a process dependent on its C-terminal transactivation domain. However, prolonged exposure under hyperosmotic conditions reduces NFAT5 activity 60.

Sodium levels similarly influence fibroblast growth factor 23 (FGF23) production in osteoblast-like cells. High sodium suppresses FGF23, while low sodium increases its synthesis. These changes inversely correlate with NFAT5 expression, and NFAT5 deletion impacts multiple genes associated with FGF23 synthesis. This underscores the regulatory role of the NFAT5-FGF23 axis in bone metabolism and related diseases 61. Hairy and enhancer of split-1 (HES1), a Notch signalling effector, is a positive regulator of NFAT5. Although HES1 is typically recognized as a transcriptional repressor, it displays a dual role in osmotic responses. ERK signalling is involved in HES1 induction, highlighting a crucial link between cellular stress and transcriptional regulation. This opens new directions for exploring how HES1 modulates NFAT5 activity, particularly in osmoprotection 62. Overall, NFAT5 integrates osmotic and non-osmotic cues, serving as a master regulator of cellular adaptation, metabolic stress pathways, and immune responses.

### Cell homeostasis

#### Osmoregulation

The kidney is an organ constantly exposed to drastic osmotic fluctuations, playing a crucial role in maintaining osmotic balance across bodily systems. In this context, NaCl induces the upregulation of both Kir1.1 potassium channels and NFAT5 at both transcriptional and protein levels in rat kidney medullary thick ascending limb (mTAL) cells. Additionally, NaCl promotes the nuclear localization of NFAT5 through ERK- and MAPK-dependent pathways 63. NFAT5 drives the expression of key osmotic-regulatory proteins, including AQP2, and its deletion results in nephrogenic diabetes insipidus 64. Moreover, increased flow combined with hyperosmolality enhances ET1 levels in MCD cells, a process that is reversed by NFAT5 inhibition via rottlerin or NFAT5 siRNA, highlighting NFAT5’s involvement in regulating renal responses to high-flow, high-osmolality conditions 65.

At the molecular level, NFAT5 function relies on its dimerization, which is crucial for phosphorylation 66, DNA encirclement, and stabilization of the NFAT5-DNA complex 67. Under normal conditions, NFAT5 maintains a nucleocytoplasmic distribution in renal medullary cells. However, during dehydration, it shifts predominantly to the nucleus, correlating with increased transcription of SMIT, further supporting its role in osmolyte accumulation 68. This nuclear translocation underscores NFAT5's role as a master regulator of renal medullary adaptation, driving the expression of stress-related proteins such as HSP70-2 to protect cells from hyperosmotic damage 69.

NFAT5 expression is also indispensable for the proper development of the kidney medulla, ensuring the activation of osmocompensatory genes essential for renal function and homeostasis 70. During embryogenesis, Na-K-2Cl cotransporter type 2 (NKCC2) precedes NFAT5 by establishing medullary hypertonicity, a prerequisite for NFAT5 osmoprotective role 71. Beyond coordinating organic osmolyte mobilization, NFAT5 induces additional molecules that support hypertonic adaptation, such as asporin, insulin-like growth factor-binding proteins (IGFBP-5 and -7), and extracellular lysophospholipase D, each contributing through distinct osmotic stress pathways 72. In MCD cells, NFAT5 promotes cell survival under hypertonic stress by upregulating RNF183, a member of the RING finger protein family, reinforcing its role in medullary adaptation 73,74.

Furthermore, NFAT5 regulates endothelin-1 (ET-1), an inhibitor of water and sodium reabsorption in MCD cells, positioning NFAT5 as a central molecule in maintaining renal sodium homeostasis 75. Serum- and glucocorticoid-inducible kinase-1 (Sgk1), expressed in the renal medulla, is regulated by extracellular tonicity through NFAT5 during dehydration-induced natriuretic states 76,77. Additionally, NFAT5 coexpresses with the secretin receptor (SCTR) in the renal cortex and medulla. SCTR contains multiple osmotic response elements (ORE) within its promoter, making its expression NFAT5-dependent 78. In kidney pathophysiology, NFAT5 expression declines alongside AQP2 and endothelial NOS (eNOS) in the renal medulla during acute kidney injury in rodent models, emphasizing the importance of spatiotemporal regulation in renal injury progression 79. Interestingly, NFAT5 appears to have a protective role in ischemia/reperfusion injury in rat kidneys, acting independently of HIF-1α 80.

NFAT5 stands out as a master regulator of kidney gene expression. Its loss triggers extensive transcriptional changes, affecting over 3000 genes in the renal cortex and more than 5,000 genes in the inner medulla, changes that are associated with renal inflammation and injury-like phenotypes 81. Moreover, a genome-wide parametric gene regulatory network analysis, based on multiomic datasets from seven human kidney samples with failed injury responses, identified NFAT5 as a key driver in the transition from healthy to maladaptive repair. This supports NFAT5 pivotal role in promoting fibrosis and chronic kidney disease progression when normal tissue repair mechanisms fail 82.

Dopamine plays a key role in inhibiting salt reabsorption in proximal tubule cells of the kidney. Aromatic l-amino acid decarboxylase (AAD), the enzyme responsible for dopamine production, is upregulated by NFAT5 under hypertonic stress, suggesting an additional layer of NFAT5-mediated control in renal osmotic homeostasis 83. Notably, NFAT5 does not regulate the ADD isoform in neural dopaminergic cell lines, indicating that its role in dopamine production is kidney-specific, with no apparent involvement in the nervous system through this pathway 84.

Beyond the kidney, high salt intake increases liver osmolality, activating NFAT5, which in turn promotes fructose production, leptin resistance, and obesity, linking NFAT5 to the pathophysiology of diabetes mellitus (DM) 85. In a hypoxic lung model, NFAT5 ablation increases oxidative phosphorylation and metabolism-related gene expression compared to wild-type cells. This identifies NFAT5 as a suppressor of mitochondrial respiration, ROS production, and oxidative gene expression, critical for limiting ROS-dependent arterial resistance in hypoxic environments 86.

In peripheral tissues, macrophages respond to interstitial sodium accumulation by upregulating NFAT5, which binds to the vascular endothelial growth factor (VEGF)C promoter, promoting its secretion. Blocking this NFAT5-VEGFC axis leads to interstitial hypertonic volume buildup, reduced eNOS expression, and elevated blood pressure, reinforcing NFAT5's role as a key osmoprotective regulator in salt-sensitive hypertension 87,88,89,90. It also acts as an osmoprotective factor in retinal pigment epithelial (ARPE-19) cells under hyperosmolar stress, boosting AR and TauT mRNA expression 91. Moreover, elevated osmolality enhances NFAT5 expression in hybridoma cells, leading to increased antibody production, highlighting its essential role in adaptive immune responses 92. NFAT5 also extends its regulatory influence to calcium handling during dehydration. It controls calcium release-activated calcium channel protein 1 (Orai1), a key component of store-operated calcium entry (SOCE) in megakaryocytes and platelets, linking NFAT5 to calcium homeostasis and coagulation processes 93,94.

NFAT5 appears to play a vital role in sodium balance during the administration of mineralocorticoid receptor antagonists. By promoting lymphatic sodium drainage, NFAT5 helps prevent sodium buildup in tissues, a key consideration in conditions like primary aldosteronism 95. In NP cells of intervertebral discs, NFAT5 responds to increased osmolality and intracellular calcium by promoting the expression of AQP2 and COX-2, enhancing cell viability and reinforcing its role as an osmoprotective factor in non-renal tissues 96,97. Interestingly, this function in NP cells is independent of primary cilia, suggesting an alternative regulatory pathway for osmotic adaptation 98. NFAT5 also plays a role in the regulation of telomerase, which consists of the telomerase reverse transcriptase (TERT), the telomerase RNA component (TERC) and the telomerase-associated protein (TEP). As the primary transcriptional activator of TERT, NFAT5 may contribute to the protection of hypertonic tissues and cells, as observed in mouse models. 99.

#### Cell proliferation, differentiation, and survival

During embryogenesis, NFAT5 is crucial for the development of the notochord and intervertebral discs, where it regulates extracellular matrix components and notochord phenotypic markers. It also modulates the sonic hedgehog (Shh) signalling pathway, further emphasizing its role in tissue patterning and maintaining structural integrity 100. Additionally, NFAT5 intersects with major developmental pathways. Its cooperation with the Wnt signalling pathway is essential for cardiomyogenesis, underscoring its role in cardiac development 101.

In this context, NFAT5 emerges as a pivotal regulator in various tissues and pathological states. A notable example is its involvement in colorectal carcinogenesis induced by Enterotoxigenic *Bacteroides fragilis* (ETBF). ETBF promotes the expression of JmjC domain-containing histone demethylase 2B (JMJD2B), a critical factor for stem cell maintenance, through NFAT5 activation, a mechanism that correlates with tumor development in the colon 102. During chondrogenesis, NFAT5 supports cartilage development by regulating the expression of SRY-box TF 9 (SOX9) under both isotonic 103 and hyperosmolar conditions 104,105. In osteoblasts exposed to high sodium chloride concentrations, NFAT5 induces the expression of osteoprotegerin (OPG) gene, which reduces osteoclastogenesis while promoting osteoblastogenesis. This highlights NFAT5 as a promising therapeutic target for high salt-induced osteopenia 106. Moreover, the activation of NFAT5 by the long noncoding RNA KCNQ1OT1, which acts as a ‘sponge’ for miR-128-3p, inhibits osteoclast differentiation in RAW 264.7 cells 107.

In reproductive physiology, NFAT5 supports osmoadaptation in bull spermatozoa, helping these cells withstand osmotic stress in the female reproductive tract, a critical factor for sperm survival and fertility 108. NFAT5 also promotes granulosa cell proliferation in the ovaries by regulating key pathways involving Wnt, β-catenin, and Bcl2, suggesting its involvement in ovarian follicle development and function 109. In muscle development, NFAT5 regulates myogenesis through its target gene Cyr61 (connective tissue growth factor), which is essential for myoblast migration and differentiation 110. NFAT5 directly binds to intronic regions of the smooth muscle α-actin (α-SMA) gene, driving smooth muscle differentiation, highlighting its influence in muscle tissue formation and repair 111. B lymphocytes exhibit a biphasic response to osmotic changes. Initially, hypertonicity increases B-cell activation and differentiation, downregulating PAX5 and upregulating CD138. However, in the second phase, cell death increases, and B-cell differentiation is reduced 112.

Additionally, NFAT5 directly promotes the expression of the L-type calcium channel gene Cacna1c by binding to its conserved TGGAAGCGTTC site, regulating cardiomyocyte maturation and cardiac electrophysiology 113. The Wnt canonical signalling pathway is abolished in more differentiated intestinal cells by the presence of NFAT5, through inhibition of the mammalian target of rapamycin (mTORC1)/Notch signalling pathway 114,115. In neonatal mouse keratinocytes, NFAT5 expression is minimal, accompanied by low levels of matrix remodelling enzymes such as metalloproteinase-3 (MMP3) and kallikrein-related peptidase 7 (Klk7). However, in adult basal keratinocytes, NFAT5 expression increases markedly, suggesting a regulatory role in epidermal matrix protease expression necessary for skin maturation and maintenance 116

NFAT5 also plays a crucial role in the NLRP3 inflammasome activity in RPE cells exposed to hyperosmotic conditions 118. Furthermore, NFAT5 is overexpressed in ARPE-19 cells under similar stress, promoting their survival 119. These findings suggest that NFAT5 plays a dual role in the retina, balancing between cell protection and potential contribution to pathological processes, as previously discussed. Additionally, hyperglycemic hyperosmolality promotes angiogenesis and retinopathy through NFAT5 activation in dermal microvascular cells 35.

Moreover, NFAT5 is implicated in corneal epithelial cell repair and nerve regeneration. Cyclosporine A triggers NFAT5 nuclear translocation, leading to increased nerve growth factor (NGF) expression at both transcript and protein levels, indicating a role in promoting corneal healing 120. Osmotic changes induced by a high-salt diet also trigger NFAT5 activation in the retina, promoting the transcription of VEGF and AQP5, along with the expression of placental growth factor (PlGF), fibroblast growth factor (FGF), and heparin-binding epidermal growth factor-like growth factor (HB-EGF), all of which are associated with neovascular pathophysiology in diseases such as age-related macular degeneration (AMD) 121,122,123. Furthermore, PlGF activates NFAT5 in endometrial stromal cells (EnSCs) independently of osmolarity, leading to the expression of downstream targets like Sgk1, HIF-1α, and VEGF-A. These findings suggest that the PlGF-NFAT5 axis plays a multifaceted role in regulating angiogenesis and trophoblast invasion through Sgk1 modulation and other signalling pathways, potentially contributing to placental pathologies 124. In the hematopoietic system, NFAT5 protects hematopoietic stem cells (HSCs) from chronic interferon type I (IFN-I) stress, highlighting its potential as a therapeutic target in hematopoietic malignancies 125.

In MCD cells exposed to 2-bromoethanamine, a nephrotoxic compound mimicking analgesic-induced nephropathy, NFAT5 fails to translocate to the nucleus under hyperosmolar stress. This prevents the induction of androgen receptor and HSP70, leading to widespread apoptosis within 48 hours, underscoring NFAT5 essential role in renal cell survival 126. NFAT5 also reduces caspase-3-mediated apoptosis in both the outer and inner renal medulla during ischemia-reperfusion injury, reinforcing its cytoprotective role in kidney cells 127.

In bone tissue, NFAT5 contributes to cementoblast differentiation by regulating miR-361-3p, a microRNA that targets NFAT5. Notably, NFAT5 knockdown mirrors the inhibitory effect of miR-361-3p overexpression, linking NFAT5 to cementogenesis 128. Cardiomyocytes exposed to the cardiotoxic chemotherapy drug doxorubicin undergo ubiquitin-independent proteasomal degradation of NFAT5, which correlates with increased cell death. Interestingly, proteasome inhibitors prevent this degradation, restoring NFAT5 levels and rescuing cardiomyocytes from apoptosis, highlighting its cytoprotective role in cardiac cells 129. Moreover, doxorubicin reduces NFAT5-dependent transcriptional activity of the TauT promoter, further linking NFAT5 to cardiomyocyte stress responses 130.

Interestingly, NFAT5 also intersects with the NFκB pathway, driving pro-apoptotic effects in human umbilical vein endothelial cells (HUVECs) under hypertonic stress by suppressing Bcl2 expression, leading to apoptosis 131. However, contradictory findings by Fedorov *et al*. revealed no significant changes in NFAT5 protein localization in HUVECs exposed to moderate NaCl-induced hyperosmolarity, suggesting that the intensity of the osmotic challenge may dictate NFAT5 activity and nuclear translocation dynamics 132.

COX-2 is essential for maintaining interstitial osmolality and cell survival in the renal medulla, and NFAT5 regulates COX-2 expression under hypertonic conditions. Inhibition of NFAT5 in canine kidney cells is associated with apoptosis, further confirming its protective role in kidney cells 133. Although hypertonicity and hyperosmolality conditions induce autophagy in certain cell types, hyperosmolality-induced autophagy appears to occur independently of NFAT5 activity 134. Instead, it is associated with acidic macrophage autolysosomal compartments 135, contributing to the defense against *E. coli* infection 136. In contrast, NFAT5 is essential for hyperosmolarity-induced autophagy in cardiomyocytes, promoting the activation of autophagy-related protein 5 (Atg5) 137. Likewise, NFAT5 protects β-pancreatic cells by preventing autophagosome formation and inhibiting β-cell death through the endoplasmic reticulum (ER) stress response 138, highlighting its diverse roles in autophagy regulation. Moreover, NFAT5 expression increases when autophagy is inhibited, inducing its target gene AQP1, which helps prevent renal damage following ischemia/reperfusion injury 139; These findings underscore the versatile transactivation properties of NFAT5 in different cellular contexts.

#### Immune response

As a master regulator of osmotic homeostasis, cell survival, and inflammation, NFAT5 is indispensable for maintaining cellular function. Its deficiency is incompatible with life, underlining its critical role in both developmental and adaptive processes. However, overactivation or loss-of-function mutations in NFAT5 are also linked to both innate and adaptive immune responses, highlighting its dual role as both a protector and a potential driver of pathological states.

In a hypertonic environment, NFAT5 promotes the transcription of CCL2, which acts as a proinflammatory activator under hyperosmotic conditions 140. Functional gene analysis and site-directed mutagenesis in NP cells have shown that NFAT5 binding to the CCL2 promoter is specifically required under hypertonic stress. In contrast, NFAT5 binding to the interleukin (IL) 6 and nitric oxide synthase (NOS2) promoters occurs independently of tonicity, suggesting that NFAT5 also supports homeostasis in intervertebral discs 141, 142.

NFAT5 plays a crucial role in antigen presentation. The major histocompatibility complex II (MHC-II), predominantly expressed by professional antigen-presenting cells (APCs) such as macrophages, dendritic cells, and B lymphocytes, relies on the transcriptional coactivator CIITA for expression. Interestingly, NFAT5 is essential for the regulation of CIITA specifically in macrophages, but not in other APCs, underscoring its unique role in macrophage activation and T-cell priming 143. In contrast, NFAT5 binds to an evolutionarily conserved promoter site of IFN-I, where it inhibits IFN-β production, promotes macrophage proinflammatory responses, and suppresses the Toll-like receptors (TLR)3 pathway 144.

Moreover, NFAT5 suppresses heme oxygenase-1 (HO-1), a stress-inducible protein, by blocking the binding of the basic leucine zipper (Nrf2) protein at its promoter region, which contributes to M1 macrophage polarization 145. In a reciprocal regulatory relationship, the HO-1 inducer hemin can inhibit NFAT5 in a model of non-alcoholic steatohepatitis, suggesting that these two molecules regulate each other under certain pathological conditions 146.

NFAT5 is essential for proper T-cell development, indicating the presence of a hyperosmolar environment within the thymus 69,147. Although NFAT5 activation is traditionally considered calcineurin-independent, in contrast to other NFAT family members, recent evidence shows that calcineurin can indeed activate NFAT5 in T cells 148. It also plays a pivotal role in adaptive immunity, supporting peripheral B-cell function in murine splenocytes under osmotic stress 149 and promoting optimal T-cell division through cyclin regulation in hypertonic conditions 150. Furthermore, CD24, a key cell surface protein essential for T-cell proliferation and homeostasis, is regulated by NFAT5 in response to osmotic stress 151, reinforcing NFAT5 involvement in adaptive immune development. Once translocated to the nucleus, NFAT5 binds DNA, activating target genes not only for osmoprotection but also for inflammation and immune responses.

lnterestingly, the role of miR-29a-3p in the differentiation and function of memory B regulatory cells (mBregs) has been demonstrated in the context of liver transplantation and acute rejection. Inhibition of miR-29a-3p leads to a significant increase in CD19+ B-cell differentiation into mBregs, enhancing their immunosuppressive capabilities through NFAT5 upregulation. Notably, this effect can be reversed by NFAT5 knockdown, confirming its essential role in this regulatory pathway. These findings suggest that targeting miR-29a-3p could offer potential therapeutic strategies to induce immune tolerance, particularly in the context of acute rejection scenarios 152.

Computational analysis has also shown that NFAT5 positively regulates IL12 synthesis by binding to the nucleosome 1 region in the IL12p40 promoter, while simultaneously inhibiting IL10 transcription by targeting the Sp1 binding site in the IL10 promoter. This dual regulation favours proinflammatory responses and supports parasite elimination by suppressing anti-inflammatory pathways 153.

## ROLE of NFAT5 IN DISEASE

**Figure 2:**
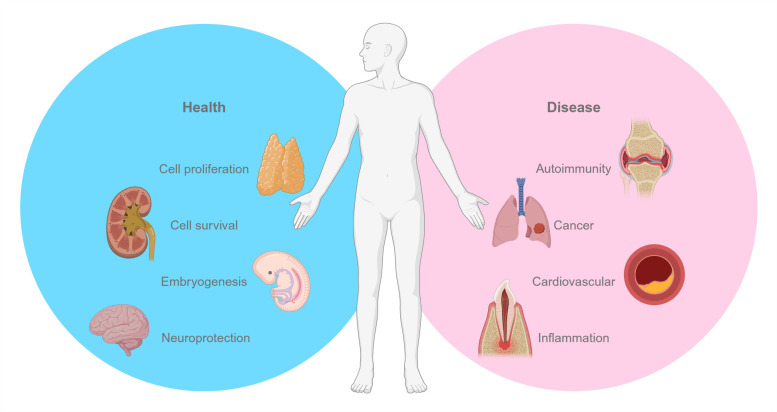
FIGURE 2: NFAT5 in health and disease. Schematic summary of the role of NFAT5 under physiological (blue) and pathological (pink) conditions. Figure created with BioRender.

NFAT5 plays a pivotal role in cellular adaptation to osmotic stress, particularly in tissues subject to significant osmolarity fluctuations, such as the kidneys. In the renal medullary region, cells endure high osmotic pressure due to urine concentration. NFAT5 acts as a protective TF in this environment by promoting the expression of osmoprotective genes, as previously discussed, helping to preserve cellular integrity and function. In the absence of NFAT5, cells fail to adapt to osmotic stress, leading to cell death and compromised kidney function. However, chronic or excessive hyperosmolar conditions, whether localized (e.g., in the kidneys, central nervous system, or eye) or systemic (such as in diabetes), can push NFAT5 regulatory capacity into a pathological state. Under these circumstances, NFAT5 activation may inadvertently contribute to inflammation and tissue damage due to its overlap with inflammatory pathways. For example, in diabetic hyperosmolar conditions, NFAT5 can drive the expression of proinflammatory cytokines and stress response genes, potentially aggravating inflammation and fibrosis in the kidneys and other tissues (**Figure 2**).

This section explores the role of NFAT5 under pathological osmotic stress conditions, highlighting how its function transitions from protective to harmful in both acute and chronic hyperosmolar environments. Understanding these dynamics may uncover potential therapeutic strategies to modulate NFAT5 activity, aiming to mitigate tissue damage in diseases associated with osmotic stress.

### Dry eye disease

Dry eye disease (DED) is characterized by a hyperosmolar tear film, which triggers inflammatory responses and cellular stress on the ocular surface. Emerging evidence suggests that NFAT5 is a key mediator in this process, regulating both protective and proinflammatory signalling pathways in corneal and conjunctival cells under hyperosmotic conditions.

A hyperosmolar state induces NFAT5 expression and nuclear translocation in human limbal epithelial cells (hLECs). This upregulation is linked to cell survival via a p38 MAPK-dependent pathway, suggesting a protective role for NFAT5 in DED 154. Similarly, corneal epithelial cells (HCE) subjected to hyperosmolar conditions exhibit increased NFAT5 and proinflammatory cytokine gene expression. Notably, genistein and calcitriol (vitamin D) suppress this expression, positioning these compounds as promising candidates for future clinical trials targeting DED treatment 155. Hyperosmotic stress also enhances NFAT5 activation and promotes IL20 secretion in HCE cells 156. Moreover, hyperosmolarity triggers the secretion of inflammatory cytokines, alarmins, and NFAT5 activation in Wong-Kilbourne derivative of Chang conjunctival (WKD) cells and HCE cells. Importantly, NFAT5 inhibition prevents the overexpression of both the chemokine CCL2 and the alarmin S100A4 in these cells 157.

Hyperosmolarity also increases CCL2 production through NFAT5 activity in modified HeLa conjunctival cells. This response is partially suppressed by cyclosporine A, dexamethasone, p38, JNK, and NFκB inhibitors 158, providing insight into the variable clinical responses observed with these treatments in DED. Additionally, a model combining hyperosmolar conditions with benzalkonium chloride exposure in conjunctival-derived cells shows upregulation of NFAT5, macrophage inhibitory factor (MIF), IL8, and CCL2 159. Furthermore, NFAT5 nuclear translocation driven by diclofenac reduces corneal surface damage without affecting tear volume, mitigating the harmful effects of hyperosmolar stress 160. Similarly, radiation-induced lacrimal gland injury triggers NFAT5 expression, contributing to the pathophysiology of DED 161. Overall, NFAT5 plays a crucial role in the adaptive response of the ocular surface to hyperosmolar stress. Given its dual role in inflammation and cell survival, NFAT5 is emerging as a potential therapeutic target for modulating inflammation and promoting cellular survival in DED.

### Diabetes

Sugar (sucrose) is a disaccharide composed of glucose and fructose, widely consumed by humans over the last two centuries. A high-glucose diet and sedentary lifestyle are associated with metabolic disorders such as obesity, metabolic syndrome, and diabetes. In high-glucose environments, NFAT5 expression is induced via p38α MAPK and PI3K activation in skeletal muscle cells. Additionally, AR and SMIT are increased in type 1 diabetes mellitus (T1DM) as an osmoprotective response 162. In healthy individuals, NFAT5 expression increases reactively in response to hyperosmotic stress in dermal biopsies, a response absents in patients with T1DM, which may explain the osmotic imbalances observed in this disease 163. These findings emphasize the differential responses to osmotic stress between healthy individuals and those with T1DM.

NFAT5 is also elevated in individuals with DM-associated dementia 164, proposing its potential as a biomarker for disease progression. Under high-glucose conditions, NFAT5 activation exacerbates renal fibrosis through AKT phosphorylation. However, lnc-ISG20-miR-486-5p binds to the 3'UTR of the NFAT5 promoter, inhibiting its expression and ameliorating diabetic nephropathy (DN) and fibrosis. This suggests that miR-486-5p acts as an NFAT5 regulator with potential therapeutic benefits for DN 165. Moreover, NFAT5 regulates AR and PKCδ expression in diabetic *in vivo* models 166, highlighting the importance of the NFAT5-AR axis in the pathophysiology of diabetes. In placental tissue from individuals with gestational diabetes, NFAT5 nuclear localization and SMIT expression increase, correlating with ceramide levels that contribute to a hyperosmotic stress environment, worsening diabetes outcomes 167.

In individuals with normal glycemia, NFAT5 correlates with IL33/ST2, while in type 2 diabetes mellitus (T2DM), it inversely correlates with body fat percentage and directly correlates with soft lean mass percentage. This indicates that NFAT5 may regulate IL33/ST2-related genes, promoting favourable metabolic outcomes 168. Furthermore, phosphorylated signal transducer and activator of transcription 3 (pSTAT3) is linked to aggravated lung injury in pulmonary tuberculosis within a T2DM rodent model. This occurs through the suppression of miR-19b and miR-1281, which upregulate NFAT5 expression 169, implying that NFAT5 plays a critical role in exacerbating T2DM-related lung complications. Finally, NFAT5 depletion in myeloid cells significantly reduces inflammation and insulin resistance in mice with high-fat diet-induced obesity 170, underscoring its potential as a target for improving metabolic outcomes in obesity and T2DM.

#### Diabetic retinopathy

In diabetic retinopathy (DR), NFAT5 overexpression is linked to retinal ganglion cell (RGC) death and NFκB activation 171. NFAT5 haplodeficiency in diabetic mice decreases the expression of PKCδ and AR in the retina compared to wild-type controls. Similarly, intravitreal NFAT5-siRNA reduces NFAT5 expression specifically in RGCs, leading to Bax (proapoptotic) downregulation and Bcl2 (antiapoptotic) upregulation, suggesting that NFAT5 inhibition may offer a therapeutic strategy for DR 166.

In RPE cells under hyperosmotic stress, the NFAT5-AR axis promotes RPE proliferation and survival 119. Additionally, NFAT5 increases the expression of osteopontin (OPN), a neuroprotective molecule, under hypertonic conditions 172. NFAT5 also induces COX-2, which in turn enhances VEGFA, IL1β, and NLRP3 transcription 173, highlighting NFAT5's dual role in promoting both retinal protection and inflammation under hyperosmolar stress. These findings suggest that NFAT5 contributes to the balance between protective and inflammatory responses in the retina.

#### Diabetic nephropathy

NFAT5 nuclear binding to OREs, along with increased AR and sorbitol dehydrogenase (SOD) levels, is observed in patients with DN. Moreover, higher NFAT5 and AR levels are associated with more severe DN phenotypes 174. NFAT5 haplodeficiency in a DN mouse model significantly reduces renal macrophages and proinflammatory cytokine expression, leading to less renal injury compared to wild-type models 175. Additionally, increased circular DNA circ0037128 and NFAT5 levels correlate with reduced microRNA-497-5p expression in kidney tissue from DN patients 176. *In vitro*, NFAT5 silencing suppresses AR expression, suggesting that NFAT5 directly regulates the AR gene in DN. The foundational study confirmed increased NFAT5/AR-dependent activity in peripheral blood mononuclear cells (PBMCs) from DN patients, reinforcing the NFAT5-AR axis role in DN pathophysiology 177. These findings further underscore the role of NFAT5 in regulating inflammation in the kidneys during DN.

### Immune-related diseases

#### Infections

NFAT5 plays a multifaceted role in inflammation, acting as both a promoter of immune responses and, paradoxically, a facilitator of viral infections. It drives the transcription of key proinflammatory cytokines like TNF-α, IL1β, and IL6, essential for recruiting immune cells and amplifying inflammation. It also upregulates chemokines such as CCL2, promoting the recruitment of monocytes, memory T cells, and dendritic cells to inflammatory sites, strengthening the initial immune defence. However, NFAT5 role is not exclusively protective, some viruses exploit its regulatory functions to enhance their replication and evade immune detection. This dual behaviour complicates its involvement in infections, as NFAT5 activation can support both immune defense and viral persistence, contributing to excessive inflammation and tissue damage.

Notably, NFAT5 acts as a host factor-viral enhancer for HIV-1 subtype viruses in HeLa CD4+ cells and THP-1 monocytes, suggesting that disrupting this interaction could inhibit viral replication and potentially slow acquired immunodeficiency syndrome (AIDS) progression 178. Furthermore, *Mycobacterium tuberculosis* triggers a positive feedback loop in HIV infection by inducing NFAT5 through TLR activation. This involves downstream signalling molecules such as MyD88 (myeloid differentiation primary response-88), IRAK1 (interleukin 1 receptor-associated kinase-1), and TRAF6 (TNF receptor-associated factor-6), ultimately exacerbating disease progression 179.

The non-structural protein 5A (NS5A) of hepatitis C virus (HCV) significantly increases NFAT5 expression, which in turn modulates HSP72 expression, enhancing HCV replication, highlighting NFAT5 as a key player in HCV propagation 180. In hepatitis B virus (HBV) infection, NFAT5 expression is downregulated due to hypermethylation of the AP1-binding site in its promoter within hepatoma cells. Additionally, HBV suppresses NFAT5 via miR-30e-5p, which targets MAP4K4. This suppression is closely associated with the development of hepatocellular carcinoma (HCC) 181. Furthermore, NFAT5 supports HCC stemness and cisplatin resistance through the ATM-NFκB pathway 182.

*In silico* analysis identified NFAT5 as a key TF regulating cytokine-regulating immune-expressed genes (CRIEGs), which contribute to the inflammatory response during COVID-19 183. Additionally, NFAT5 controls the expression of EAAT3, promoting glutamate uptake and increasing intracellular glutathione, a vital antioxidant that protects cells from Epstein-Barr virus (EBV)-induced oxidative stress 184.

Coxsackievirus B3 (CVB3), responsible for myocarditis, produces the protease 2A, which cleaves NFAT5 at Gly503, generating an inactive 70 kDa dominant-negative form of the protein. This promotes viral replication by impairing NFAT5 function, representing a viral evasion mechanism 185. Moreover, *Porphyromonas gingivalis*, induces miR-132 in THP-1 cells, which targets and suppresses NFAT5, reducing TNF-α production. This suggests that *P. gingivalis* uses NFAT5 inhibition as an immune evasion strategy 186.

NFAT5 also plays a crucial role in defence against *Leishmania major*, primarily by activating TLRs and iNOS in macrophages, a process dependent on inhibitor of κB kinase (IKK)-β activity 187. Interestingly, TLR activation through lipopolysaccharide (LPS) in RAW 264.7 mouse macrophages triggers xanthine oxidase-ROS production, a response inhibited by high salt through ROS mitochondrial suppression, suggesting hypertonicity and inflammation counterbalance NFAT5 activation 188. Moreover, low LPS doses in macrophages efficiently recruit NFκB p65 and c-Fos to proinflammatory genes. In contrast, high LPS doses lead to NFAT5-independent NFκB recruitment. Notably, H3K27me3 demethylation emerges as an NFAT5-dependent early mechanism that promotes p65 recruitment to TLR4-induced proinflammatory gene promoters 189.

Under hypertonic stress, NFAT5 enhances macrophage production of nitric oxide (NO), supporting the immune response and serving as a protective barrier against the parasite 190. On the other hand, under a high-salt diet, NFAT5 is induced by glucocorticoids in neutrophils, which paradoxically impairs their antibacterial function and reduces their ability to defend against *Listeria monocytogenes* infection 191.

#### Chronic Inflammation

Beyond its well-established role in osmoregulation, NFAT5 plays a significant role in modulating inflammatory responses, particularly by regulating key cytokines and chemokines. This positions NFAT5 as a central player in both localized and systemic inflammation. Dysregulation of NFAT5 activity has been implicated in chronic inflammatory diseases, where persistent activation exacerbates tissue damage. This section examines how NFAT5 influences immune responses, contributes to inflammatory pathologies, and interacts with other immune-related molecular pathways, highlighting its potential as a therapeutic target for inflammatory conditions.

Patients undergoing peritoneal dialysis are frequently subjected to chronic inflammation. Dialysis fluids enriched with glucose, mannitol, or NaCl activate NFAT5 in mesothelial cells, promoting CCL2 production, a crucial step in the fibrosis pathway 192,193. Furthermore, NFAT5 expression is notably upregulated in peritoneal biopsies from uremic patients, accompanied by an increased frequency of CD68+ activated macrophages. This suggests an active role for NFAT5 in peritoneal inflammation and fibrotic progression 194.

NFAT5 exerts dual roles in CD4+ T cells, adapting its function depending on the microenvironment. Under hyperosmolar stress, NFAT5 promotes IL2 production and the expression of Th17-associated genes such as RORγt and IL23R. In contrast, activation via anti-CD3 antibody skews the response toward IFNγ and IL17, while inhibiting the Treg response. Notably, in an experimental colitis model, NFAT5 deficiency leads to a more severe inflammatory response, underscoring its critical regulatory role in intestinal inflammation 195.

NFAT5 also plays a pivotal role in sepsis by binding to the TNFα promoter, interacting with NFκB p65, and recruiting the p300 subunit, thereby enhancing LPS-induced inflammation 196. Notably, NFκB binds to the NFAT5 promoter to enhance the expression of glycolysis-related genes and proinflammatory cytokines. This interaction is vital for restoring metabolic activity in immune-tolerant macrophages during sepsis 197. NFAT5 dysfunction is observed in sepsis, with reduced expression of target genes essential for urine concentration, including ClC-K1, barttin, UTA-1, and AQP2, partially explaining the urinary imbalances observed in acute kidney injury 198. Additionally, in a murine sepsis model, NFAT5 expression decreases alongside a rise in M2 macrophages, a process associated with miR-223 regulation, a key factor in IL4-driven M2 polarization, indicating that NFAT5 regulates anti-inflammatory macrophage responses 199.

Under hyperosmolar conditions, invariant NKT (iNKT) cells, which typically produce both IL4 and IFNγ, lose the ability to produce IFNγ but retain IL4 synthesis when stimulated via TCR, IL12, or IL18. This NFAT5-dependent response highlights its significance in chronic inflammatory diseases such as rheumatoid arthritis (RA) 200. Serum amyloid A (SAA) induces NFAT5 expression through TLR2/4-dependent pathways, promoting macrophage infiltration and arthritis progression in mice. Notably, inhibiting either NFAT5 or TLR2/4 reverses these effects, highlighting NFAT5 role in inflammatory arthritis 201.

Hyperosmolar stress enhances NFAT5 nuclear translocation in primary human chondrocytes and the ATDC5 chondrocyte cell line, supporting its involvement in cartilage inflammation and survival 202. IL1β is upregulated in chondrocytes from individuals with osteoarthritis (OA), driving a proinflammatory state via an NFAT5-SIRT-dependent pathway, similar to the effects of melatonin. NFAT5 inhibition reduces the production of TNFα, IL1β, prostaglandin E2 (PGE2), and NO in chondrocytes, highlighting NFAT5 as a key player in OA pathogenesis and melatonin as a potential therapeutic modulator 203. In a knee OA model induced by medial meniscus destabilization, NFAT5 expression is upregulated, and mice with genetic NFAT5 disruption show reduced synovial inflammation and cartilage damage. This is likely due to a decrease in CCL2, IL1β, MMP-13, and ADAMTS-5 production, as well as a reduction in monocyte/macrophage recruitment 204. NFAT5 expression is significantly elevated in cartilage samples from OA patients, contributing to metalloproteinase overexpression through TLR2 activation by 29 kDa fibronectin fragments 205. Moreover, synovial cells sense mechanical stimuli similarly to osmotic stress, activating NFAT5, suggesting that NFAT5 plays a crucial role in cellular adaptation to mechanical changes in OA 206.

In NP cells, TNFα drives NFAT5 nuclear translocation, regulating proinflammatory chemokines such as CXCL1, CXCL2, and CXCL3. Interestingly, this regulation depends on a conserved NFκB-binding site rather than the predicted NFAT5-binding site. This underscores the indirect yet essential role of NFAT5 in driving the pathophysiology of intervertebral disk degeneration (IDD) through NFκB 207. In the spinal dorsal horn (SDH), NFAT5 triggers inflammation exclusively in astrocytes and regulates AQP4 expression via Aurora kinase B (AURKB)-mediated phosphorylation, contributing to neuropathic pain development 208.

In a formalin-induced inflammatory pain model, NFAT5-heterozygous mice exhibit reduced pain sensitivity compared to wild-type mice. These mice also show lower expression of c-Fos, p-ERK, and phosphorylated NMDA receptor subunit 2B (p-NR2B), molecules regulated by the mTOR pathway, positioning NFAT5 as a potential therapeutic target for inflammatory pain 209.

In a mouse model of perforating corneal injury (PCI), NFAT5 is highly upregulated in recruited corneal macrophages. Depleting NFAT5 in myeloid cells accelerates corneal oedema resolution, indicating that NFAT5 plays a critical role in corneal inflammation 210. In lens epithelial cells, UV-B radiation increases NFAT5 expression and NFκB activation, especially in the HLE-B3 cell line, suggesting a collaborative role of both factors in cataractogenesis 211. Moreover, transgenic mice expressing a dominant-negative NFAT5 protein exhibit impaired lens development and develop nuclear cataracts shortly after birth 212.

In Duchenne muscular dystrophy (DMD), a chronic idiopathic inflammatory myopathy, NFAT5, SMIT, AR, and TauT are overexpressed compared to controls, implicating this osmoregulatory-proinflammatory pathway in chronic muscle inflammation 213. Notably, NFAT5 predominantly localizes to the nucleus in DMD muscle cells. Unlike other cell types, its activity remains unaffected by hyperosmolar conditions or proinflammatory cytokines such as IFNγ, IL1β, and TNF-α, suggesting that permanent fibrosis in DMD may lock NFAT5 in an active state, contributing to reduced cell viability 214, 215. High NaCl levels are observed in the skin of atopic patients, where high-salt conditions drive Th2 polarization via the NFAT5-Sgk1 pathway 216. Interestingly, high salt also induces an anti-inflammatory Th17 phenotype, promoting Foxp3 and IL17A expression in CD4+ T cells 217.

NFAT5 is crucial for salt-induced differentiation of CD4+ T cells into effector phenotypes, and silencing NFAT5 significantly impairs the cytotoxic activity and antitumor efficiency of these cells 218. In comparison, macrophages expressing NFAT5 are more prone to polarize toward a Th1 proinflammatory phenotype, characterized by increased IL12, Fizz-1, and arginase 1 expression, compared to Lewis lung carcinoma and ID8 ovarian carcinoma cells. This proinflammatory role was further confirmed *in vivo* through adoptive transfer models, where NFAT5-deficient macrophages displayed reduced antitumor activity 219. Inhibition of NFAT5 improves allograft survival in a murine heart transplantation model. Treatment with KRN2 attenuates acute allograft rejection by suppressing T cell activation and promoting Treg cell differentiation, suggesting that NFAT5 modulation could be a promising approach for transplant tolerance 220.

In LPS-induced rodent nephrotic proteinuria, NFAT5 and NFκB are upregulated in both *in vivo* and LPS-incubated podocytes. Interestingly, NFAT5 inhibition suppresses NFκB activation and ameliorates nephrotic proteinuria, supporting its role in renal inflammation 161. Similarly, in a seawater inhalation-induced acute lung injury model, NFAT5 is upregulated in lung tissue and alveolar macrophages, while NFκB activity diminishes when NFAT5 and proinflammatory cytokines are silenced, highlighting NFAT5's involvement in lung injury pathophysiology 221.

Silencing NFAT5 in myeloid cells reduces osteoclastic activity, leading to slower orthodontic tooth movement, less periodontal bone loss, lower root resorption, and preserved bone density under high-salt conditions 222,223. In periodontitis, NFAT5 gene expression is downregulated compared to healthy gingival tissue, accompanied by upregulation of miRNA-20a, miRNA-30e, and miRNA-93, suggesting a potential post-transcriptional regulatory mechanism 224.

#### Autoimmunity

NFAT5 plays a pivotal role in both innate and adaptive immunity, making it a key regulator in various autoimmune disorders. This section explores the connection between NFAT5 and autoimmunity, highlighting its involvement in disease pathogenesis and potential therapeutic implications.

Mutations in NFAT5 have been linked to primary immune regulatory disorders, particularly autoimmune lymphoproliferative syndromes 225. Additionally, pathogenic NFAT5 variants have been identified in 14 families with familial autoimmunity, encompassing primary Sjögren's syndrome (pSS), systemic lupus erythematosus (SLE), and RA, underscoring its role in genetic predisposition to autoimmunity 226. NFAT5 protein expression is elevated in renal biopsies from lupus nephritis (LN) patients, correlating with increased inflammatory cytokine expression and proteinuria severity. In a pristane-induced SLE mouse model, myeloid-specific NFAT5 deficiency prevented the development of SLE and LN, highlighting its critical role in disease progression 227.

NFAT5 also regulates the G0/G1 switch gene 2 (G0S2) via the p1 promoter site. In myasthenia gravis (MG), tacrolimus, an immunosuppressant, reduces NFAT5 and G0S2 expression in PBMCs, providing insights into both disease mechanisms and tacrolimus' mode of action 228. Furthermore, in high-salt environments, NFAT5 activation promotes Th17 differentiation, exacerbating autoimmune encephalomyelitis 229. In MG and thymoma-associated MG, miR-20b is downregulated, leading to increased NFAT5 expression. Given that miR-20b directly targets NFAT5, its downregulation may act as a tumor suppressor mechanism, potentially explaining thymoma progression in MG patients 230.

Interestingly, NFAT5 haplodeficiency alleviates experimental autoimmune encephalomyelitis (EAE) but only in female mice. This protective effect is associated with an increased regulatory T cells (Treg) population in the central nervous system (CNS) and spleen, as well as a notable reduction in CD11c+CD8α+ dendritic cells, specifically in the female CNS 231.

In Behcet disease, oral manifestations are associated with NFAT5 downregulation 232. Additionally, transfection of miR-18b, miR-106a, and miR-363-3p into CD4+ T cells suppresses Rorc, Rora, IL17A, and IL17F expression, thereby inhibiting Th17-driven IL17 production by blocking Rora- and NFAT5-mediated transcriptional activation 233. In active vitiligo, both Foxp3 and NFAT5 transcripts are significantly downregulated, suggesting a potential link between NFAT5 deficiency and Treg dysfunction 234. Similarly, in recurrent Graves' disease, NFAT5 expression is diminished in CD4+ T cells, indicating that its downregulation may contribute to disease relapse 235. These findings emphasize NFAT5 as a crucial modulator of autoimmunity. For an extensive review on NFAT5 in autoimmune diseases, refer to Lee and colleagues 236.

In T1DM, NFAT5 plays a role in Treg dysfunction. miR-181a-driven NFAT5 activation impairs Treg differentiation, contributing to immune imbalance. Notably, blocking either miR-181a or NFAT5 restores Treg development and reduces autoimmune activity in pancreatic islets, suggesting a potential therapeutic target 237. Additionally, autoantibodies against NFAT5 have been reported in PES1, a syndrome in which NFAT5 dysfunction contributes to tubulointerstitial nephritis due to its role in regulating the AQP2 promoter 238.

NFAT5 is also implicated in RA, where its expression is elevated in synovial tissue, promoting synovial proliferation and angiogenesis 239. In RA macrophages, NFAT5 enhances cell survival and CCL2 secretion, contributing to chronic inflammation by promoting macrophage resistance to apoptosis 240. In murine models of arthritis, myeloid-specific NFAT5 deletion reduces disease severity, dendritic cell maturation, and the differentiation of pathogenic Th1/Th17 cells, further highlighting its role in pro-inflammatory immune responses 241. Interestingly, in collagen-induced arthritis models, NFAT5 expression is significantly reduced in mice on a low-salt diet, correlating with decreased arthritis severity compared to those on normal or high-salt diets. This supports the role of NFAT5 in linking osmotic stress with inflammation in autoimmune diseases 242.

In fibroblast-like synoviocytes (FLSs), IL1β and TGF-β induce CCL2 and coagulation factor III secretion via p38 MAPK-activated NFAT5. Inhibiting NFAT5 prevents lamellipodia formation, cell migration, and invasion—processes that can be partially restored in RA-FLSs upon CCL2 and IL1β stimulation. Additionally, NFAT5 enhances CCL20 and CXCL8 transcription in RA synovial fibroblasts (RASFs) upon exposure to neutrophil-derived lactoferrin 243. Notably, KRN2, an inhibitor of TGF-β-induced FLS migration, underscores NFAT5’s role in driving inflammation in RA pathogenesis 244. However, paradoxically, NFAT5 overexpression suppresses RA-FLS proliferation and invasion—an effect reversed by miR-338-5p co-transfection 245. These findings suggest that NFAT5 may have dual roles in autoimmune diseases, acting as both a pro-inflammatory regulator and a context-dependent modulator of immune responses.

### Cancer

Numerous factors contribute to the development and progression of neoplasms, including impaired immunity, decreased immune surveillance, increased proliferation of neoplastic cells, and chronic inflammation. This section explores the role of NFAT5 in cancer initiation and progression, shedding light on its complex functions in various cancer types.

In lung adenocarcinoma (LUAD) cells, NFAT5 expression increases alongside AQP5. Inhibition of both molecules reduces proliferation and migration, while NFAT5 overexpression enhances AQP5 expression, fostering tumor cell growth 246. However, bioinformatics analysis revealed that NFAT5 expression was significantly decreased in LUAD and lung squamous cell carcinoma (LUSC). Interestingly, high NFAT5 expression correlates with better overall survival in LUAD patients but worse survival in LUSC patients, highlighting the context-dependent functions of NFAT5 in different lung cancer subtypes 247. In laryngeal squamous cell carcinoma (LSCC), the lncRNA small nucleolar RNA host gene 16 (SNHG16), a putative oncogene, shows elevated expression in both cells and tissue. SNHG16 binds to miR-140-5p, whose overexpression inhibits LSCC cell proliferation and migration. Notably, NFAT5 is a direct target of miR-140-5p, and its downregulation suppresses the Wnt/β-catenin signalling pathway 248.

In EGFR-mutated non-small cell lung cancer (NSCLC), macrophage-conditioned medium enhances cell migration and resistance to tyrosine kinase inhibitors (TKIs), highlighting the critical role of the tumor microenvironment in cancer progression. Suppressing NFAT5 expression reduces both cell migration and resistance to gefitinib, an EGFR inhibitor, underscoring NFAT5's potential as a therapeutic target 249. Circular RNA circ_0001944 is highly expressed in NSCLC and correlates with poor prognosis. This RNA sponges miR-142-5p, a negative regulator of NFAT5, leading to NFAT5 overexpression. This cascade enhances proliferation, migration, invasion, and glycolysis in NSCLC cells 250. Similarly, lncRNA MFI2-AS1, enriched in NSCLC-derived exosomes, sponges miR-107, increasing NFAT5 expression and promoting tumor progression 251. Furthermore, serum exosomes from NSCLC patients contain circCCDC134, which supports growth, metastasis, and glycolysis by absorbing miR-625-5p, resulting in NFAT5 upregulation 252. Macrophages in the tumor microenvironment induce NFAT5 expression in A549 LUAD cells, contributing to cisplatin resistance, cell migration, and invasion. siRNA silencing of NFAT5 reverses these effects, reducing resistance and metastatic potential 253.

NFAT5 also plays a significant role in platinum-resistant epithelial ovarian carcinoma. Overexpression of RBMS3 protein inhibits β-catenin/CBP signalling by stabilizing several negative regulators, including NFAT5, through competitive inhibition of miR-126-5p-mediated repression 254. Basal NFAT5 expression is notably elevated in epithelial ovarian cancer cell lines (ES-2, OVCAR3, TOV112D, and UWB1.289), while NFAT5 silencing reduces viability, proliferation, and migration, particularly in UWB1.289 cells. Moreover, increased cytoplasmic NFAT5 expression in ovarian cancer specimens is associated with more favourable clinical and pathological outcomes 255. These observations suggest that NFAT5 may serve as a prognostic biomarker in ovarian cancer, reflecting its involvement in both tumor progression and patient outcomes.

Furthermore, NFAT5 plays a pivotal role in endometrial cancer, where the NFAT5-COX-2 signalling axis is critical for tumor progression. NFAT5 is more abundant in high-grade tumors and modulates the expression of key genes, including COX-2 and HIF1α, suggesting an intricate interplay that may drive cancer progression 256.

Integrin α6β4 clustering, in the presence of chemoattractants, enhances NFAT5 transcriptional activity, promoting the migration of human breast carcinoma cells 66,257. NFAT5 is proposed as a biomarker for inflammatory breast cancer, with potential as a screening tool for breast tumors 258,259,260. In MCF-7 breast cancer cells, NFAT5 synergizes with STAT3, directing the inflammatory response toward IL17 and VEGFA production, supporting tumor inflammation and angiogenesis 261. Additionally, NFAT5 regulates S100A4, an essential protein linked to tumor metastasis, through an integrin-dependent mechanism 262. Overexpression of NFAT5 is observed in inflammatory mammary carcinoma and vascular-invasive mammary carcinoma, where it activates the noncanonical Wnt pathway, associated with poor prognosis 263.

The role of NFAT5 in HCC remains controversial; NFAT5 functions as a tumor suppressor by promoting PARP-1- and Bax/Bcl-2-dependent apoptosis. It also inhibits the epithelial-mesenchymal transition (EMT), downregulating claudin-1 and fibronectin, key markers of invasion and metastasis 264. The mitochondrial aspartyl-tRNA synthase 2 (DARS2) functions as an oncogene in HCC, promoting cell proliferation and inhibiting cell death. NFAT5 binds to DARS2, suppressing its expression and thereby supporting tumor suppression 181. In contrast, NFAT5 expression is reported to be 93% higher in HCC tumors, independent of their aetiology. This elevated NFAT5 expression is associated with recurrence, metastasis, and mortality, likely through COX-2 signalling 265. These conflicting findings highlight the need for further research to clarify the precise role of NFAT5 in HCC tumorigenesis.

NFAT5 expression is elevated in chronic lymphocytic leukaemia (CLL), promoting proliferation and resistance to apoptosis. Depletion of NFAT5 leads to cell cycle arrest and enhanced apoptosis. AQP5, is also regulated by NFAT5 in CLL cells, further supporting its role in cell survival 266. Upstream Stimulatory Factor 2 (USF2) promotes CLL progression by inducing NFAT5 ubiquitination and suppressing STIP1 homology and U-Box protein 1 (STUB1), a tumor suppressor 267. In acute lymphoblastic leukaemia cells, purple sweet potato anthocyanins inhibit NFAT5 activity, inducing calcicoptosis, a calcium overload-driven cell death mechanism 268. Under hyperosmolar conditions, NFAT5 drives the activation of paired box 2 (PAX2) in coordination with PAX5, in pre-B acute lymphoblastic leukaemia cells 269.

In glioblastoma multiforme (GBM), NFAT5 expression is markedly increased in both tumor samples and GBM cell lines, positively correlating with the WHO-GBM classification. NFAT5 regulates the angiogenic activity of the long noncoding RNAs SBF2 antisense RNA 1 (SBF2-AS1) and miR-338-3p 270, supporting neoplastic cell survival and promoting angiogenesis. Additionally, miR-641 levels, significantly lower in GBM tissues than in controls, negatively regulate NFAT5 expression and transactivation. This regulation affects the p-AKT signalling pathway, ultimately promoting GBM cell survival 271. Furthermore, circFOXO3 acts as a competing endogenous RNA, enhancing NFAT5 expression via miR-138-5p and miR-432-5p 272, highlighting the importance of NFAT5 in GBM promotion and survival.

NFAT5 is implicated in colorectal brain metastasis, marking its relevance in distant tumor spread 273**. **In colon cancer cells under hypertonic stress, S100A4 is upregulated by NFAT5 binding to the ORE of S100A4, located in the first intron region, dependent on its methylation status 274. Interestingly, in a colorectal cancer model, NFAT5 expression is reduced in circulating tumor cells, suggesting its potential role in circulating tumor dynamics 275. Additionally, NFAT5 has been proposed as a progression biomarker in colon cancer, identified in a competitive endogenous RNA network 276.

NFAT5 promotes oral squamous cell carcinoma (OSCC) cell proliferation by enhancing EGFR N-glycosylation, which facilitates its translocation to the plasma membrane under hypertonic conditions, a process essential for OSCC survival 277. Under hyperosmotic conditions, NFAT5 also promotes the expression of Ranbp3l, a protein whose deficiency is associated with a cancer-promoting phenotype in human renal cell carcinomas 278. In pancreatic ductal adenocarcinoma (PDAC), NFAT5 is similarly upregulated and linked to poor prognosis. Phosphoglycerate kinase 1 (PGK-1), a key glycolytic enzyme involved in ATP generation, is identified as an NFAT5 target in PDAC 279.

NFAT5 is overexpressed in a cohort of 25 patients with adrenocortical carcinoma, where ten exhibited NFAT5 amplification and overexpression, confirmed by qPCR. This pattern correlates with high sensitivity and specificity for tumor malignancy 280. In melanoma, elevated levels of the long noncoding RNA myocardial infarction-associated transcript (MIAT) are linked to poorer patient outcomes. Mechanistic studies reveal that MIAT enhances NFAT5 transcription by recruiting TCF12 to the NFAT5 promoter, promoting melanoma cell proliferation, migration, and invasion 281.

The lncRNA AP000842.3 is a negative regulator of NFAT5, contributing to cuproptosis in prostate adenocarcinoma and promoting malignant progression. NFAT5 expression is partially modulated by miR-206, indicating a complex regulatory network involved in prostate cancer development, where NFAT5 plays a central role 282. A high-salt diet induces activation of the NLRP3 inflammasome complex via NFAT5, leading to CD4+ T-cell-mediated immune-related adverse events 283. NFAT5 is highly expressed in exhausted tumor-induced CD8+ T cells and is associated with decreased tumor control. Notably, NFAT5 deletion improves tumor control by downregulating exhaustion-related proteins such as HMG-box TF (TOX) and programmed cell death protein 1 (PD-1), while enhancing the expression of key cytokines such as IFN-ɣ and TNFα 284.

NFAT5 interacts with PARP-1 to prevent R-loop accumulation and subsequent DNA damage in osteosarcoma cells 285. Moreover, NFAT5 recruits the methyl transferase METTL3 to R-loops, facilitating RNA methylation via m6A, which promotes R-loop resolution 286. RNA helicases DDX5 and DDX17 function as transcriptional coactivators of NFAT5, promoting tumor cell migration by activating NFAT5 target genes 287. These helicases interact with CDK-9, enhancing neoplastic cell proliferation, positioning NFAT5 as a key regulator of tumor growth and invasiveness 288.

This highlights the multifaceted role of NFAT5 in cancer, influencing immune evasion, cell survival, proliferation, inflammation, and metastasis. A deeper understanding of the molecular mechanisms underlying NFAT5 involvement in these processes could uncover new therapeutic strategies aimed at targeting NFAT5 to restrain cancer progression. Further investigations are essential to clarify NFAT5 function across different cancer types and to explore how its modulation might yield therapeutic benefits.

### Cardiovascular diseases

Beyond its well-established role in renal regulation of systemic blood pressure, NFAT5 has emerged as a crucial extrarenal regulator, influencing vascular smooth muscle cells (VSMCs) in arteries and arterioles. These cells are essential in maintaining vascular tone, and NFAT5 supports their adaptive responses under osmotic and mechanical stress, conditions frequently encountered in the vasculature, particularly in hypertension. In arterial hypertension, chronic mechanical stress on blood vessels activates NFAT5, which promotes the expression of genes associated with vascular remodelling, inflammation, and fibrosis. While these responses initially help preserve vascular integrity, sustained NFAT5 activation eventually drives maladaptive changes, contributing to vascular stiffening, increased peripheral resistance, and even atherosclerotic plaque formation. This pathological shift highlights the dual role of NFAT5, it facilitates early adaptation but, over time, contributes to long-term vascular deterioration in chronic hypertension.

In this section, we will explore the emerging role of NFAT5 in the extrarenal regulation of blood pressure and its implications for arterial hypertension and cardiovascular disease. Prolonged NFAT5 activation may exacerbate vascular dysfunction, transforming what begins as a protective mechanism into a contributor to disease progression. From a therapeutic perspective, targeting NFAT5 pathways in VSMCs presents a promising opportunity to address these maladaptive processes.

NFAT5 also plays a critical role in cardiac osmoregulation. Under hypertonic stress, NFAT5 activates its target genes, promoting adaptive cellular responses, while hypotonic environments suppress its expression. This osmoregulatory capacity is particularly vital during myocardial infarction, where cellular hydration and stress responses are crucial for cardiac cell survival 289, 290. Additionally, high serum sodium levels promote thrombosis and vascular events, and NFAT5 contributes to this process by enhancing the synthesis and secretion of von Willebrand factor (vWF) from endothelial cells, thus linking sodium imbalance with prothrombotic events 291.

Associations between NFAT5 and blood pressure regulation have been identified in large cohorts of individuals of European ancestry, particularly concerning elevated pulse pressure 292. Notably, the minor G allele of rs9980 in the NFAT5 locus on chromosome 16 is strongly associated with increased plasma sodium concentrations across European, Asian, and Indian populations 293. Furthermore, the NFAT5-VEGFC signalling axis plays a pivotal role in maintaining systemic osmotic balance and blood pressure regulation. Systemic depletion of interstitial mononuclear phagocytes, accompanied by NFAT5-VEGFC downregulation, promotes salt-sensitive hypertension, underscoring NFAT5 role in preserving vascular homeostasis under hyperosmolar stress 294.

In systemic arterial hypertension, NFAT5 is upregulated in VSMCs through a c-Jun-dependent pathway in response to mechanical stretching of the vessel wall 295. This activation results in the nuclear accumulation of the NFAT5c isoform, contributing to vascular remodelling and adaptive changes in arterial stiffness 296. Interestingly, genetic ablation of NFAT5 specifically in VSMCs disrupts the balance of extracellular matrix proteins, such as actin β-like 2 (ACTB2), tenascin 2 (TNC), and COX-2, which leads to maladaptive arterial remodelling 297.

Moreover, NFAT5 deficiency in VSMCs leads to the formation of lipid-rich aortic lesions, characterized by lipid droplet accumulation in the subintimal layer, a hallmark of early atherogenesis. Notably, NFAT5 regulates lipid metabolism-related genes in response to cholesterol overload, suggesting a protective role against atherosclerosis in hyperlipidaemic conditions 298. Moreover, NFAT5 activation is regulated through the ERK1/2 pathway. This activation drives CCL2 expression in infiltrating monocytes, promoting collateral artery formation in murine models of hind limb ischemia 299. Additionally, NFAT5 enhances arteriosclerosis via NLRP3 inflammasome activation in endothelial cells, contributing to chronic vascular damage 300.

High-salt diets are strongly associated with the development of hypertension, a major risk factor for neovascular AMD. In this context, hyperosmotic stress triggers NFAT5 activation in RPE cells, promoting the expression of VEGF, AQP5, and AQP8, a combination that may worsen retinal damage and oedema 301. Moreover, high-salt environments amplify proinflammatory cytokine production, including IL6 and CCL2, particularly after LPS stimulation in ARPE-19 cells in an NFAT5-dependent manner 302.

The NFAT5-VEGFC-lymphangiogenesis axis also plays a crucial role in human arterial hypertension 303. In a Wistar rat hypertension model, increased nucleic acid-binding activity of Annexin-A2, alongside a rise in NFAT5 transactivation activity without altering its abundance, occurs alongside an increase of AQP2 in the MCD 304. These findings suggest that Annexin-A2 modulates NFAT5 activity, contributing to the kidney role in systemic hypertension.

Furthermore, studies in spontaneous hypertensive rodent models show increased NFAT5 expression, co-expressed with lymphopoiesis markers such as prospero homeobox-1 (Prox-1), lymphatic vessel endothelial hyaluronan receptor 1 (Lyve-1), podoplanin (POD), and VEGFC in the left ventricle. These changes coincide with hemodynamic disturbances, including impaired diastolic function, positioning NFAT5 as a key player in cardiac muscle lymphatic-dependent remodelling 305.

The importance of NFAT5 extends to heart development, as it is essential for normal cardiac morphogenesis during embryogenesis. This is highlighted by the lethality observed in NFAT5(-/-) homozygous models, with most *in vivo* studies relying on haplodeficient NFAT5 models to circumvent early lethality 306. In dialysis patients, severe arterial calcification is linked to increased NFAT5 expression and decreased miR-381-3p levels. miR-381-3p directly binds to the 3' UTR of NFAT5, acting as a negative regulator. This interaction suppresses apoptosis and slows vascular calcification, suggesting a potential therapeutic target for chronic kidney disease management 307. In atherogenesis, NFAT5 promotes inflammation by inducing CCL2 expression in monocytes, facilitating macrophage migration, a key step in plaque formation driven by BMCs 308.

NFAT5 also contributes to obesity and insulin resistance through white fat epigenetic suppression, with a correlation observed between NFAT5 expression in subcutaneous adipocytes and body mass index 309. Moreover, AQP1 and NFAT5 co-expression has been observed 310 alongside elevated proinflammatory and remodelling molecules, such as F-actin and α-SMA, in models of aortic stiffness linked to diabetes and hypercholesterolemia 311.

A high-salt diet also exacerbates cardiovascular risk by promoting plasminogen activator inhibitor-1 (PAI-1) expression in ApoE-/- mice. This effect is NFAT5-dependent, as the PAI-1 promoter contains a TGGAATTATTT NFAT5 binding site, enhancing antifibrinolytic activity in endothelial cells, a mechanism that could contribute to prothrombotic states and vascular dysfunction 312.

In the context of viral myocarditis, NFAT5 plays a protective role. Infections with Coxsackievirus B3 (CVB3), known for inducing cardiac damage, are exacerbated under NFAT5 deficiency. Mice lacking NFAT5 exhibit higher viral loads, worsened cardiac pathology, and reduced survival rates. These findings highlight NFAT5's dual function, enhancing the antiviral immune response while preserving cardiomyocyte integrity. NFAT5 deficiency also disrupts cytokine signalling, reducing levels of IFNβ1, CXCL10, and IL6, which weakens the host immune defence. Furthermore, NFAT5 deficiency impairs stress granule formation, compromising cardiomyocyte structural stability through the reduction of plakophilin-2 levels, a key component of desmosomal junctions. These findings position NFAT5 not only as a stress regulator but also as a potential therapeutic target to mitigate CVB3-induced cardiomyopathy, offering a novel perspective on cardioprotection during viral infections 313.

NFAT5 has emerged as a prosurvival molecule in cardiotoxic environments, particularly under chemotherapy-induced stress. For example, in the context of doxorubicin exposure, known for its cardiotoxic effects, NFAT5 supports myocyte survival. Interestingly, doxorubicin enhances NFAT5 degradation through a ubiquitin-independent pathway, suggesting that NFAT5 acts as a protective regulator during chemotherapeutic stress 129. These results highlight the importance of NFAT5 in cardiovascular disease pathophysiology.

### Neurological diseases

Osmoadaptation in the brain is essential for maintaining cellular and systemic homeostasis, particularly under conditions of fluctuating osmotic pressure. This process involves specialized mechanisms that enable brain cells to adapt to changes in osmolality, protecting them from dehydration or swelling, both of which can compromise cellular integrity and function. Regions such as the hypothalamus, which regulate systemic osmolality by controlling thirst, water intake, and hormonal responses, rely heavily on osmoadaptation for survival. This section will explore the role of NFAT5 in brain osmoadaptation and neuroinflammation, highlighting its potential therapeutic applications.

NFAT5 has emerged as a key therapeutic target in neuroinflammation and blood-brain barrier (BBB) protection. In a kainic acid-induced seizure model, NFAT5 haplodeficiency resulted in reduced BBB leakage, indicating its potential in preventing seizure-induced neurovascular damage 314. Similarly, NFAT5 haplodeficiency mitigates neuroinflammation in a high-fat diet/streptozotocin-induced diabetic model, with a significant decrease in ionized calcium-binding adapter molecule 1 (Iba-1) immunoreactivity in the hippocampus compared to wild-type controls 315. This evidence underscores NFAT5's protective role in neuroinflammatory contexts. Additionally, NFAT5 expression was found to be increased in CHME5 microglia subjected to oxygen-glucose deprivation/reoxygenation, while the overexpression of miR‑374a‑5p contributed to the polarization of microglia from a proinflammatory (M1) to an anti-inflammatory (M2) state, indicating a complex regulation of NFAT5 in microglial activation 316.

During hypoxia/ischemia, NFAT5 and HIF-1α inversely regulate NKCC1 expression in hippocampal neurons, suggesting that these two molecules have complementary homeostatic roles in maintaining tissue integrity under stress 317. Furthermore, in a rat ischemia/reperfusion injury model, NFAT5 overexpression promoted astrocyte survival, inhibited apoptosis, and reduced histone acetylation, thereby supporting neurogenesis and enhancing Nrf2 nuclear transport 318. These findings highlight the multifaceted role of NFAT5 in preserving neuronal function and facilitating tissue remodelling during neuroinflammatory and ischemic conditions.

NFAT5 expression is also critical in the hypothalamus, where it is present in pro-opiomelanocortin (POMC) neurons. Its expression increases following systemic TNFα administration, and NFAT5+/- mice exhibit a blunted proinflammatory response to TNFα, characterized by inhibited POMC expression, reduced anorexia, and hyperthermia. These findings underscore NFAT5's role as a mediator of systemic inflammation through the hypothalamic axis 319. In a mouse stroke model induced by middle cerebral artery occlusion, NFAT5 was induced only in the ipsilateral hemisphere, with peak expression observed 72 hours after cerebral lesion induction 320. This suggests a significant involvement of NFAT5 in ischemic brain injury. Moreover, under ischemic/hypoxic conditions, NFAT5 and its downstream SMIT gene product help protect neurons from oxidative stress, further supporting its neuroprotective role 321.

NFAT5 is not considered an early-response gene, such as Atf3, Verge, or Klf4, but rather functions as a delayed-response TF. Immunohistochemistry studies show that NFAT5 translation peaks 90 minutes after systemic hypertonicity, indicating that preliminary signalling events precede its activation 322. NFAT5 is also upregulated in OX-42-positive microglia during transient middle cerebral artery occlusion (MCAO) and LPS injection in the substantia nigra, which mirrors findings from primary microglia cultures exposed to LPS, IFNγ, and IL4. These conditions induce NFAT5 expression, emphasizing its involvement in inflammatory microglial activation 323.

Additionally, NFAT5 plays a central role in age-related microglial activation and cognitive decline. In aged mice, hippocampal NFAT5 expression and microglial activation are significantly elevated compared to young mice. Notably, NFAT5 haploinsufficiency reduces microglial activation and mitigates cognitive impairment in middle-aged mice, positioning NFAT5 as a key driver of neuroinflammatory changes associated with aging 324. In Parkinson's disease, NFAT5 emerges as a potential therapeutic target. LPS-stimulated BV-2 cells mimic Parkinson's disease-associated microglial inflammation, with miR-29c inhibiting NLRP3 inflammasome activation by targeting NFAT5. This suggests that NFAT5 contributes to the progression of Parkinson's disease 325, linking it to neuroinflammation and cognitive decline 326.

Furthermore, hypernatremia, a condition characterized by elevated sodium levels, is associated with central nervous system dysfunction and can lead to demyelinating lesions, similar to those observed in osmotic demyelination syndrome (ODS). Both acute (6 or 24 hours) and chronic (over 7 days) hypernatremia conditions increase NFAT5-associated NOS2 expression and NO production in microglia, which is correlated with intracellular calcium dynamics. These findings shed light on how hypernatremia affects microglial activation and identify potential therapeutic targets for neuroinflammatory diseases such as NFAT5 327. On the other hand, hyponatremia (low sodium levels) also modulates NFAT5-dependent NO production in microglia, contributing to neuronal dysfunctions observed during rapid-to-chronic sodium corrections, such as in ODS 328.

Interestingly, the exposure of pregnant rats to hyperosmotic solution results in increased levels of IL17, TNFα, NGF and NFAT5 in the brains of their offspring, which is associated with autism-like behaviors 329. In a rat model of epilepsy, both NFAT5 and the lncRNA X-inactive-specific transcript (XIST) are found to be upregulated. XIST functions as a sponge for miR-29c-3p, which normally inhibits NFAT5. By sequestering miR-29c-3p, XIST prevents the regulation of NFAT5, leading to increased inflammation and glutamate accumulation in astrocytes 330. NFAT5 is also significantly upregulated in an oxygen-glucose deprivation/reoxygenation astrocyte model, with this upregulation mediated by circCELF1, highlighting its involvement in ischemic brain injury 331.

**Figure 3:**
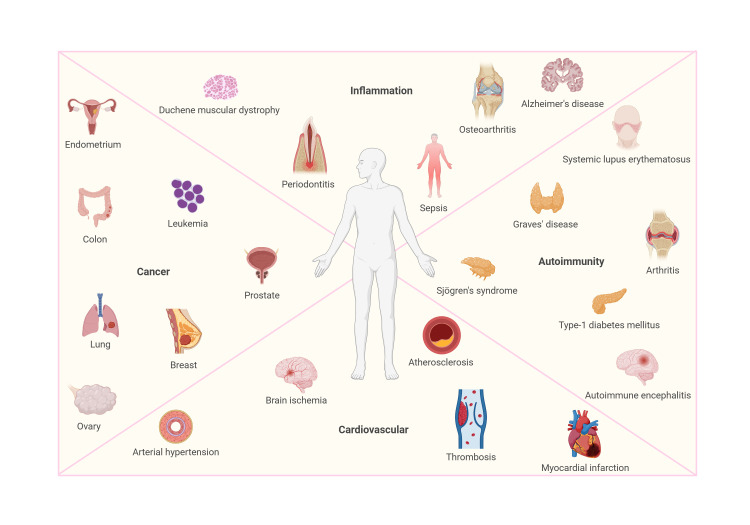
FIGURE 3: Involvement of NFAT5 in various pathological conditions. NFAT5 activity is implicated in the development of inflammatory diseases, autoimmune disorders, cardiovascular conditions and cancer. Figure created with BioRender.

In infants with severely abnormal neurodevelopment, NFAT5 transcript levels are significantly elevated, which correlates with poor outcomes in neonatal hypoxic-ischemic encephalopathy. This suggests that NFAT5 could serve as a biomarker for neurodevelopmental impairment, offering potential for early detection and intervention strategies 332. Additionally, NFAT5 is implicated in stress-related disorders. A strong correlation between Cacnac1C and NFAT5 has been observed in the amygdala of both mice and humans experiencing chronic stress, positioning NFAT5 as a potential therapeutic target for psychiatric conditions associated with chronic stress 333. Furthermore, NFAT5 plays a critical role in bupivacaine (BUP)-induced neurotoxicity. Following BUP exposure, levels of lncRNA OIP5-AS1 and NFAT5 decrease, while miR-34b levels increase, leading to reduced neuronal proliferation and heightened apoptosis in dorsal root ganglion neurons. OIP5-AS1 acts as a spacer for miR-34b, enabling upregulation of NFAT5. The addition of NFAT5 counteracts the negative effects of miR-34b, promoting neuronal survival and highlighting its therapeutic potential in neurotoxicity scenarios 334. This further supports NFAT5 role in modulating neuroinflammation in various neurological conditions (**Figure 3**).

### NFAT5 regulation and therapeutic approaches

After exploring the different NFAT5 activators, this section shifts focus to NFAT5 inhibitors, providing a detailed analysis of the tightly controlled regulatory mechanisms that modulate its activation. Understanding these inhibitors is essential for uncovering potential therapeutic strategies that could target NFAT5 in various diseases.

Conjugated linoleic acid has been shown to downregulate NFAT5 expression in subcutaneous abdominal adipose tissue after 4 weeks of supplementation. This suggests a negative regulatory role of NFAT5 in adipose metabolism and obesity, indicating its involvement in metabolic processes beyond its traditional role in osmotic stress response 335. Similarly, the activation of AMPK suppresses NFAT5 in renal medullary interstitial cells (RMICs) under hyperosmotic stress, leading to reduced NFκB nuclear translocation and COX-2 expression, which promotes apoptosis. This underscores the crucial role of NFAT5 in regulating renal cell survival under stress conditions 336. Furthermore, Metformin, a widely used drug for T2DM, inhibits NFAT5 under hypertonic conditions, thereby reducing the expression of osmoprotective genes like AR and BGT1. This raises concerns for DM patients with renal disease, as NFAT5 activity is critical for cellular adaptation to osmotic stress 337. Dexmedetomidine, an α2-adrenergic receptor agonist, also inhibits both NFAT5 and SIRT protein expression in a diabetic hyperglycemia-ischemia/reperfusion model, indirectly supporting NFAT5's role in diabetes-associated neurovascular damage 64.

In addition to these pharmacological inhibitors, microRNAs also play a pivotal role in modulating NFAT5 expression. For instance, miR-568 suppresses NFAT5 in CD4+ T cells and Treg cells, leading to reduced activation (CD25, CD69, CD154), decreased IL2 production, and diminished T-cell proliferation. This highlights a direct role for NFAT5 in lymphocyte regulation 338. Likewise, miR-10b-5p inhibits NFAT5 by targeting its 3’-UTR region in C2C12 myoblasts, impairing cell differentiation, which points to NFAT5’s involvement in muscle cell maturation 339. Similarly, Roquin 1 inhibits NFAT5 via translational inhibition in MEFs and CD4+ T cells 340.

NFAT5 induction and NFAT5-dependent transcription are inhibited by cyclosporine A and FK506 in a TCR-dependent manner in T lymphocytes. However, their induction by hyperosmotic stimuli is not blocked by calcineurin. Moreover, osmotic stress response genes, such as AR, are not induced upon T-cell activation, suggesting distinct mechanisms regulating NFAT5 transcriptional functions 148. Additionally, B lymphocyte-induced maturation protein-1 (Blimp-1) represses NFAT5 activity during cell maturation in corneocytes and in morphologically abnormal cornified layers, suggesting its role in skin physiology 116. Glycerol has also been shown to reduce NFAT5 and IL1β expression in HaCaT keratinocytes under hypertonic stress, suggesting a cytoprotective role by modulating inflammatory responses in the skin 341.

NFAT5 is not only involved in osmotic stress but also in cancer biology 257. It plays a critical role in tumor progression, metastasis, and recurrence, as well as prognosis following surgical resection in NSCLC 269,342. miR-194 binds to the 3’-UTR region of NFAT5, reducing NFAT5 expression and protein abundance in high-glucose-induced NSCLC cells, linking metabolic dysregulation to tumor progression 343. In a similar context, miR-211, a known tumor suppressor in metastatic melanoma, has been described as a suppressor of NFAT5 344. *In vivo* studies using a C57BL/6 mouse model injected with B16BL6-NFAT5-knockdown melanoma cells demonstrated weak melanoma tumor growth and a decrease in lung and liver nodule formation, further supporting the role of NFAT5 in melanoma growth and metastasis 345.

Additionally, urea pre-treatment has been found to inhibit hypertonicity-induced changes in the expression of the physiological effector gene AR, highlighting a molecular mechanism through which urea modulates tonicity-dependent signalling and underscores the role of NFAT5 in regulating gene transcription 68. The small molecules KRN2 and KRN5, which exhibit high oral bioavailability and metabolic stability, have been shown to ameliorate experimentally induced arthritis in mice without serious adverse effects, decreasing proinflammatory cytokine production. Notably, orally administered KRN5 was more effective than methotrexate, a commonly used antirheumatic drug, demonstrating better potency and safety. These findings suggest that KRN2 and KRN5 could be promising therapeutic agents for the treatment of chronic arthritis 346.

At the molecular level, NFAT5 is regulated by phosphorylation, with phosphatases such as SHP-1 interacting directly with Thr143 of the NFAT5 regulatory site, inhibiting its nuclear translocation 347. A high-salt environment attenuates SHP-1 inhibitory effect on NFAT5 activation through the inhibition of protein targeting to glycogen (PTG), revealing the regulatory mechanism of SHP1 on NFAT5 under hypertonic conditions 348. Furthermore, lipid droplet-associated protein fat-specific protein-27 (FSP27) inhibits NFAT5 nuclear translocation and represses CCL2 expression, suggesting an important role of NFAT5 in lipid metabolism and inflammation, beyond its osmoprotective function 349. Gonadotropin-releasing hormone (GnRH) agonists inhibit NFAT5 expression in leiomyoma cells at pharmacological doses 350. Similarly, the selective progesterone receptor modulator ulipristal acetate (UPA) inhibits NFAT5 concomitantly with a decrease in versican, aggrecan, and brevican proteoglycans, leading to a significant decrease in leiomyoma tissue 351.

Similarly, the transcriptional coactivator TAZ, highly expressed in the kidney, inhibits NFAT5 activity through tyrosine phosphorylation, suggesting another layer of regulatory control in kidney physiology 352. Additionally, NFAT5 is a target of miR-223, whose upregulation inhibits platelet-derived growth factor-BB (PDGF-BB)-induced motility and proliferation of human aortic smooth cells 353. In addition, miR-96-5p upregulation inhibits angiotensin-stimulated VSMC proliferation and migration by targeting NFAT5 354.

Exosomes derived from miR-146a-5p-enriched bone marrow mesenchymal stem cells (MSCs) inhibit NFAT5 and M1 polarization of microglia in an intracerebral haemorrhage (ICH) model in rats, accompanied by reductions in CCL2, COX2, and iNOS levels, suggesting that NFAT5 is a potential target for ICH treatment 355. Moreover, exosomes derived from MSCs inhibit Th17 polarization through NFAT5 inhibition via miR-1246 and alleviate inflammation in periodontitis 356.

Intermittent hypoxia and reoxygenation, processes related to severe sleep apnoea, lead to a decrease in miR-21-5p and miR-23-3p expression in a TLR4-dependent manner in PBMCs from patients with obstructive sleep apnea. This is associated with increased cytotoxicity, apoptosis, and elevated NFAT5 gene expression, among other hypoxia- and proinflammatory-related genes. These effects are reduced by miR-21-5p mimic transfection 357.

Computational approaches have also contributed to identifying potential NFAT5 inhibitors. For example, molecular dynamics simulations have pinpointed a peptide that may inhibit NFAT5 dimerization at its DNA-binding domain, although further validation in biological and clinical settings is needed 358. Additionally, PARP-1 and heat shock protein 90 (HSP90) have been shown to modulate NFAT5 expression in the HEK293 cell line, with PARP-1 rescuing NFAT5 transcriptional activity and HSP90 enhancing its activity and maintaining protein stability 359. These findings point to a complex regulatory network controlling NFAT5 expression. Computational analysis also revealed putative quadruplex-forming sequences in TF binding sites, including NFAT5, suggesting that G-quadruplex formation could influence NFAT5's ability to regulate gene transcription 360. NFAT5 regulation involves post-translational modifications, protein-protein interactions, and subcellular localization. Its nuclear translocation is mediated by specific signalling pathways and nuclear transport proteins.

NFAT5 is tightly regulated at the transcriptional level by the RNA helicases DDX5 and DDX17, which enhance the inclusion of NFAT5 exon 5. This exon contains a premature translation termination codon, leading to the degradation of NFAT5 mRNA via the nonsense-mediated decay (NMD) pathway and ultimately reducing NFAT5 protein levels 287. Additionally, NKCC2A regulates NFAT5 expression and transcriptional activity in mTAL cells under hypertonic conditions 361. In these cells, an inflammatory response triggered by elevated urinary TNFα under hyperosmolar stress is coordinated by NFAT5 and NKCC2A, highlighting their interplay in osmotic regulation and inflammation 362. NFAT5 nucleocytoplasmic trafficking is controlled by casein kinase 1 (CK1), which phosphorylates NFAT5 at Ser158, followed by phosphorylation at Ser155, as observed in HeLa cells 363.

**Figure 4:**
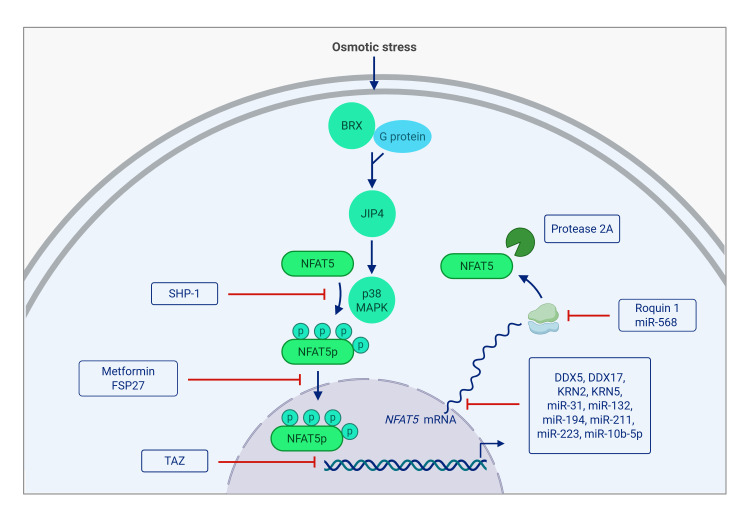
FIGURE 4: Regulators of NFAT5 through its activation pathway. NFAT5 activity can be regulated at different stages of its activation and synthesis. The Src homology region 2 domain-containing phosphatase-1 (SHP-1) dephosphorylates NFAT5p at regulatory site Thr143 inhibiting its capacity to migrate into the nucleus. Metformin and lipid droplet-associated protein fat-specific protein-27 (FSP27) treatment inhibits nuclear translocation of NFAT5p. Transcriptional coactivator with PDZ-binding motif (TAZ) suppresses DNA-binding and transcriptional activities of NFAT5p. DEAD-box helicase (DDX) 5 and 17, KRN2 and 5, and the microRNAs miR-31, miR-132, miR-194, miR-211, miR-223, and miR-10b-5p reduce the gene expression of NFAT5. The miRNA-binding protein Roquin 1 and miR-568 suppress NFAT5 translation by directly binding to the 3'-UTR untranslated region of NFAT5. Moreover, protease 2A, produced by Coxsackievirus B3, cleaves NFAT5 at Gly503 promoting viral replication. Figure created with BioRender.

At the post-transcriptional level, NFAT5 expression is further regulated by various non-coding RNAs. miR-31 downregulates NFAT5, increasing NP cell viability and reducing cell death, which is associated with protection against intervertebral disc degeneration 364. Similarly, the lncRNA MIAT regulates NFAT5 by sponging miR-613 in LPS-stimulated microglia 365. Additionally, miR-20b inhibits NFAT5 and inactivates the TLR signalling pathway by preventing TLR2-TLR4 dimer formation, thereby reducing inflammation in alveolar type II epithelial cells following *M. tuberculosis* infection 366. These findings highlight the diverse molecular mechanisms that regulate NFAT5 expression and activity. The ability to modulate NFAT5 levels through these pathways presents potential therapeutic strategies for conditions associated with NFAT5 overactivation (**Figure 4**).

## CONCLUSION

NFAT5, a versatile TF, is an important regulator of cellular responses to osmotic stress and other stimuli. It plays a critical role in maintaining cellular homeostasis by activating genes involved in osmoprotection, inflammation and cell survival. As a protector during hyperosmotic changes, NFAT5 regulates the expression of genes involved in osmoprotection, such as aquaporins, osmolyte transporters and heat shock proteins. The numerous triggers include osmotic stress such as changes in extracellular osmolality, inflammatory stimuli such as various cytokines and other inflammatory signals, and mechanical forces as they act on cells and tissues. In inflammation, NFAT5 is able to induce the expression of pro-inflammatory cytokines and thus contribute to the inflammatory response. This TF plays an essential role in proliferation and differentiation and regulates cell proliferation, differentiation and migration, processes that control tissue development and repair. However, its role in cancer progression, tumor growth and metastasis has been extensively studied. Understanding the intricate mechanisms of NFAT5 activation and its downstream targets offers potential therapeutic opportunities. Targeting NFAT5 could offer new strategies for the treatment of various diseases, including kidney disease, autoimmune diseases, diabetes, blood disorders, cancer and brain diseases.

## AUTHOR CONTRIBUTION

Alfredo Domínguez-López and Fátima Sofía Magaña-Guerrero performed the literature search, wrote the manuscript. Alfredo Do-míguez-López, Fátima Sofía Magaña-Guerrero, Beatriz Buentello-Volante, Óscar Vivanco-Rojas and Yonathan Garfias wrote the manuscript. Alfredo Domínguez-López and Yonathan Garfias revi-sed the manuscript. Yonathan Garfias conceived and supervised the review, wrote and revised the manuscript. All the authors have read and approved the final version of the manuscript.

## CONFLICT OF INTEREST

The authors declare no conflicts of interest.
